# Prenatal air pollution influences neurodevelopment and behavior in autism spectrum disorder by modulating mitochondrial physiology

**DOI:** 10.1038/s41380-020-00885-2

**Published:** 2020-09-22

**Authors:** Richard E. Frye, Janet Cakir, Shannon Rose, Leanna Delhey, Sirish C. Bennuri, Marie Tippett, Stepan Melnyk, S. Jill James, Raymond F. Palmer, Christine Austin, Paul Curtin, Manish Arora

**Affiliations:** 1 Barrow Neurological Institute at Phoenix Children’s Hospital, Phoenix, AZ USA; 2 North Carolina State University, Raleigh, NC USA; 3 Arkansas Children’s Research Institute, Little Rock, AR USA; 4 Department of Pediatrics, University of Arkansas for Medical Sciences, Little Rock, AR USA; 5 College of Public Health, University of Arkansas for Medical Sciences, Little Rock, AR USA; 6 Department of Family and Community Medicine, University of Texas Health Science Center, San Antonio, TX USA; 7 Department of Environmental Medicine and Public Health, Icahn School of Medicine at Mount Sinai, New York, NY USA

**Keywords:** Physiology, Biochemistry, Predictive markers, Autism spectrum disorders

## Abstract

We investigate the role of the mitochondrion, an organelle highly sensitive to environmental agents, in the influence of prenatal air pollution exposure on neurodevelopment and behavior in 96 children with autism spectrum disorder (ASD) [45 with neurodevelopmental regression (NDR); 76% Male; mean (SD) age 10 y 9 m (3 y 9 m)]. Mitochondrial function was assessed using the Seahorse XFe96 in fresh peripheral blood mononuclear cells. Second and third trimester average and maximal daily exposure to fine air particulate matter of diameter ≤2.5 µm (PM_2.5_) was obtained from the Environmental Protection Agency’s Air Quality System. Neurodevelopment was measured using the Vineland Adaptive Behavior Scale 2nd edition and behavior was assessed using the Aberrant Behavior Checklist and Social Responsiveness Scale. Prenatal PM_2.5_ exposure influenced mitochondrial respiration during childhood, but this relationship was different for those with (*r* = 0.25–0.40) and without (*r* = −0.07 to −0.19) NDR. Mediation analysis found that mitochondrial respiration linked to energy production accounted for 25% (SD = 2%) and 10% (SD = 2%) of the effect of average prenatal PM_2.5_ exposure on neurodevelopment and behavioral symptoms, respectively. Structural equation models estimated that PM_2.5_ and mitochondrial respiration accounted for 34% (SD = 4%) and 36% (SD = 3%) of the effect on neurodevelopment, respectively, and that behavior was indirectly influenced by mitochondrial respiration through neurodevelopment but directly influenced by prenatal PM_2.5_. Our results suggest that prenatal exposure to PM_2.5_ disrupts neurodevelopment and behavior through complex mechanisms, including long-term changes in mitochondrial respiration and that patterns of early development need to be considered when studying the influence of environmental agents on neurodevelopmental outcomes.

## Introduction

Autism spectrum disorder (ASD) is a behaviorally defined disorder [1] which may affect as many as 1 in 45 children in the United States [2]. Recent estimates suggest that inherited single gene and chromosomal defects account for a minority of ASD cases [3] and that ASD most likely arises from a complicated interaction between genetic predisposition and environmental exposures [4, 5].

Fine air particulate matter of diameter 2.5 µm or less (PM_2.5_) is an important measurable environmental toxicant that is derived from multiple ubiquitous sources. Short-term and long-term PM_2.5_ exposure is linked to many chronic diseases, including pulmonary and cardiovascular disease [6]. Air pollution, including PM_2.5_ exposure, during gestation and early life increases the risk of developing ASD [7]. Although environmental agents such as PM_2.5_ are epidemiologically associated with ASD, the biological mechanisms which mitigate their effects are poorly understood [8].

One of the critical gaps in knowledge is how an environmental exposure can result in disease when the symptoms arise years after the exposure. Mitochondrial metabolism is a promising mechanism through which the environment can cause long-term effects since mitochondria undergo long-term adaptive changes in physiology as a result of environmental stressors through a process known as mitoplasticity [9]. Studies support the notion that air pollution alters mitochondrial physiology. For example, air pollution, particularly PM_2.5_, can alter mitochondrial DNA (mtDNA) [10–12] and prenatal air pollution exposure is linked to mitochondrial-derived peptides in cord blood that are associated with long-term changes in mitochondrial physiology [13]. Prenatal mitochondrial function is important for brain development as a recent animal study has shown that mitochondrial dysfunction during gestation alters white matter brain connectivity [14].

The possibility that mitochondria are involved in the pathophysiology of environmentally influenced disease is particularly compelling because of the high prevalence of mitochondrial dysfunction in ASD. Indeed, 30–50% of children with ASD demonstrate biomarkers of mitochondrial dysfunction [15, 16] and up to 80% show abnormal electron transport chain (ETC) activity in lymphocytes and granulocytes [17, 18] as well as in post-mortem brain [19]. Interestingly the great majority of cases of mitochondrial disease in individuals with ASD do not show mutations in mitochondrial or nuclear mitochondrial genes, suggesting that alterations in mitochondrial function could be the result of adaptive changes caused by environmental exposures [15]. Alternatively, alterations in non-mitochondrial genes not uncommonly associated with ASD such as PTEN [20] and mTOR [21, 22] can alter mitochondrial function, potentially contributing to mitochondrial dysfunction and predisposing the mitochondrial to be sensitive to environmental agents.

Individuals with mitochondrial dysfunction are susceptible to environmental triggers, sometimes resulting in neurodevelopmental regression (NDR). Shoffner et al. [23] showed that the majority of children with ASD and mitochondrial disease developed ASD symptoms after a sudden rapid NDR; the NDR found to be commonly associated with a fever. In about one-third of the cases, the fever was associated with routine vaccination suggesting that any association between vaccination and NDR was caused by the fever [23]. Individuals with mitochondrial disease are known to experience NDR with illness [24], and a meta-analysis found that NDR was more common in children with ASD and mitochondrial disease than in ASD children without mitochondrial disease [15]. A recent study suggests that those with ASD and NDR may have unique abnormalities in mitochondrial physiology as compared to those with ASD without NDR [25]. Thus, NDR may be a hallmark of abnormal mitochondrial physiology in ASD. This is consistent with our in vitro lymphoblastoid cell line (LCL) model of ASD where a subset of LCLs with mitochondrial dysfunction are found to be very sensitive to increases in physiological stress [26–32]. Thus, in this study we focus on the reported history of NDR since the subset of children with ASD and NDR may have underlying abnormalities in mitochondrial physiology that create vulnerabilities to environmental stressors.

We hypothesize that prenatal exposure to PM_2.5_ drives changes in neurodevelopment and behavior through its effect on the mitochondria. Mitochondrial respiration was measured using the Seahorse XFe96 analyzer which provides four major indices of mitochondrial respiration [25]. As a first step, the relationship between prenatal air pollution exposure, as measured by average (^ave^PM_2.5_) and maximal (^max^PM_2.5_) PM_2.5_ exposure during gestation, and these four indices of mitochondrial respiration are investigated, particularly with respect to NDR. We hypothesize that prenatal PM_2.5_ exposure has long-lasting effects on mitochondria which can be measured in childhood–long after the prenatal exposure, particularly in those with a history of NDR [33].

If air pollution is found to affect mitochondrial function, then the question remains whether the effect of air pollution on neurodevelopment and behavior: (a) can be explained by the effect of air pollution on the mitochondria, (b) is a direct effect unrelated to the mitochondria, or (c) a combination of both. Thus, as a second step, we investigated this possibility with the hypothesis that the mitochondrial, at least in part, is involved in the effect of prenatal PM_2.5_ exposure on neurodevelopment and behavior. This was investigated using two analysis methods. Using mediation analysis, the effect of PM_2.5_ on mitochondrial respiration is shown to partially account for the relationship between PM_2.5_ and neurodevelopment and behavior. Using structural equation models (SEMs) the mutual and overlapping influences of mitochondrial respiration and prenatal PM_2.5_ exposure on both neurodevelopment and behavior is demonstrated in individuals with ASD. SEMs allow for the consideration of more complex relationships than the linear models including considering the important influence of redox metabolism and the interaction between mitochondrial parameters. This study is the first to question whether prenatal exposure to PM_2.5_ could result in a long-term change in mitochondrial respiration and whether such changes can account for eventual variations in behavior and neurodevelopment  in children with ASD. Demonstrating that some of the effects of PM_2.5_ exposure is mediated through the mitochondria opens the door to developing targeted treatments that can protect the mitochondria and/or optimize mitochondrial function during the prenatal periods and potentially prevent the development of ASD.

## Material and methods

### Participants

Protocols used in this study were registered in clinicaltrials.gov as NCT02000284 and NCT02003170 and approved by the Institutional Review Board at the University of Arkansas for Medical Sciences (Little Rock, AR). Parents of participants provided written informed consent.

Exclusion criteria were (i) chronic treatment with medications that would detrimentally affect mitochondrial function such as antipsychotic medications; (ii) vitamin or mineral supplementation exceeding the recommended daily allowance, and (iii) prematurity.

Inclusion criteria for participants included the ability to tolerate phlebotomy and, for those with ASD, a diagnosis of ASD. The ASD diagnosis was documented by one of the following: (i) a gold-standard diagnostic instrument such as the Autism Diagnostic Observation Schedule and/or Autism Diagnostic Interview-Revised (ADI-R); (ii) the state of Arkansas diagnostic standard, defined as agreement of a physician, psychologist and speech therapist who specializes in ASD; and/or (iii) Diagnostic Statistical Manual diagnosis by a physician along with standardized validated questionnaires which have good correspondence to the gold-standard instruments including the Social Responsiveness Scale (SRS) [34, 35], the Social Communication Questionnaire [36–38] and the Autism Symptoms Questionnaire [39] and diagnosis confirmation by the Principal Investigator (first author) who specializes in the diagnosis and treatment of children with ASD. In our recent clinical trial, [40] methods (ii) and (iii) were validated by re-evaluating a portion of the participants diagnosed with methods (ii) and (iii) using the ADI-R and finding that their ADI-R scores fell well within the diagnostic criteria for ASD.

Children underwent a fasting blood draw in the morning. Thus, this study reports physiological and environmental measurements on a total of 102 children without known mitochondrial disease, 96 diagnosed with ASD, 3 typically developing (TD) siblings of children with ASD, and 3 TD children without  siblings  with ASD. This was a subset of a  larger cohort of a study of mitochondrial function in children with ASD which included those families who provided birth residence information. Some children provided repeated blood samples for analysis: 78 provided 1 sample (74 ASD and 4 TD), 22 (20 ASD and 2 Sibs) provided 2 samples, and 2 (2 ASD) provided 3 samples. TD individuals did not have any neurological disorders or developmental delays and ASD symptoms were ruled out with a Social Communication Questionnaire score <12.

The NDR history was obtained using the Developmental and Neurobehavioral Regression (DANR) questionnaire which has been developed as part of our ASD research program. The DANR records detailed information about NDR including specific questions on premorbid functioning before the regression, duration of the regression, specific skills lost and when the skills were regained, whether there was a single or multiple regressions and any known trigger such as illness, fever, or seizure (See Supplementary Fig. S1). The responses are reviewed to ensure that any symptoms of regression are consistent with NDR typical of ASD. For example, NDR in a child with ASD following the ASD diagnosis (e.g., at 8 years of age, 5 years following the ASD diagnosis) would not be consistent with an ASD associated NDR. While other types of NDR may occur in children with ASD, this study is focused on NDR as part of the evolving ASD symptoms.

**Fig. 1:**
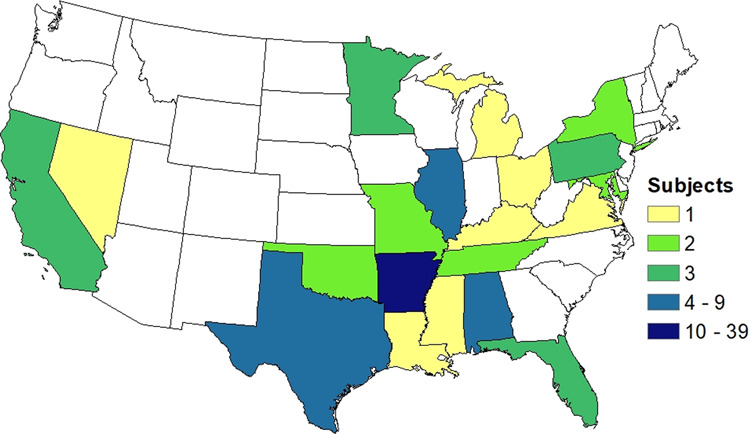
Geographic distribution of study participants. States in which participants lived during gestation are highlighted in color corresponding to the number of participants from that state.

Supplementary Table S1 outlines the participant characteristics stratified by NDR using our standard methodology [40]. This study did have a larger percentage of children with regression (47%) than would be expected from the general ASD population. There was an imbalance in the proportion of females across groups but no significant differences in other characteristics, including age, were found across ASD groups. Figure 1 shows the continental U.S. geographic distribution of participants.

In general, several personnel specializing in specific part of the study  were used to maintain blinding to the outcome measures when possible. Specifically, the blood and relevant clinical information was collected by different people (LD, MT) from the personnel who performed the mitochondrial respiratory and oxidative stress laboratory measurements (SR, SCB, SM) and air pollution analysis (JC). Data from all sources was compiled by non-laboratory staff (LD, MT) and statistical analysis was performed by individuals not directly involved in sample collection or laboratory analysis (JC, REF).

### Behavioral measurements

As standard practice for our laboratory [40, 41], research staff were trained by a multispecialty team consisting of two licensed psychologists and a speech therapist prior to performing assessments. During the study, a research psychologist supervised research staff and provided feedback and retraining if necessary. The Vineland Adaptive Behavior Scale (VABS) 2nd edition was completed using the Survey Interview Form and parents completed the Aberrant Behavior Checklist (ABC) and the SRS. These are common validated measures for providing an assessment of neurodevelopmental function and ASD symptoms in children with ASD [40, 41].

The ABC is a 58-item questionnaire that measures disruptive behaviors and has convergent and divergent validity. The SRS is a 65-item questionnaire that measures the severity of social skill deficits across five domains which has been shown to have good correspondence to the gold-standard instrument [34, 35]. The VABS is a reliable and valid measure of the ability to perform age-appropriate everyday skills, including communication, daily living, social and motor skills, through a 20–30 min structured interview with a caretaker. Since behavior measures (i.e., SRS, ABC) were found to depend on neurodevelopment, as measured by the VABS Adaptive Behavioral Composite, in our previous studies [40, 41], VABS was used as a covariate in the analyses examining SRS and ABC scores.

### PM_2.5_ exposure

Parents provided the zip code for the participant’s residence at birth on a standard medical history intake form. Regional daily PM_2.5_ measures were obtained from the Environmental Protection Agency ‘s Air Quality System (AQS) similar to other studies investigating ASD and PM_2.5_ exposure [42]. Although some previous studies have modeled PM_2.5_ using geospatial modeling such as land use regression analysis, such models are best utilized when the population studied is in a relatively circumscribed area where monitors are sparse. In this study, the participants lived across the entire United States, mostly in urban areas where air monitors are relatively dense and the participants themselves are relatively widely separated. Thus, it is likely that using the monitor in closest proximity to the participant is more accurate than imputing estimates of exposures. Thus, data were obtained using the Environmental Protection Agency’s data download tool: “AirData: Air Quality Data Collected at Outdoor Monitors Across the US.” Using the interactive map of air quality monitors, the PM_2.5_ monitor closest to the participant’s birth zip code that was active using the participants birth year was located. If multiple monitors were equivalently close to the center of the zip code, a monitor either east or west was preferred due to the general east to westerly direction of wind in the US. We derive both the maximum (^max^PM_2.5_) and average (^ave^PM_2.5_) PM_2.5_ exposure in the 6 months prior to birth (i.e., second and third trimesters). The first trimester was not examined since environmental agents reach the fetus most efficiently through the placenta and the placenta is not fully formed until the beginning of the second trimester.

### Blood collection and processing

Up to 20 ml of blood was collected into an ethylenediaminetetraacetic acid (EDTA)-Vacutainer tube, chilled on ice, and centrifuged at 1500 × *g* for 10 min at 4 °C to separate plasma within 30 min of collection. Plasma was removed and stored at −80^o^C for later analysis. Plasma was replaced with room temperature wash buffer containing Ca^+2^/Mg^+2^-free PBS with 0.1% BSA and 2 mM EDTA. Diluted blood was then layered on top of Histopaque-1077 (Sigma Aldrich, St. Louis, MO, USA) and centrifuged at 400 g for 30 min at room temperature. Peripheral blood mononuclear cells (PBMCs) were washed twice with wash buffer and counted using a hemocytometer. Viability exceeded 95% in all cases and recovery was ~10^6^/ml in most cases. Isolation procedure duration was 90–120 min. Fresh PBMCs were used for the mitochondrial respiration assay.

### Mitochondrial respiration assay

Bioenergetic data was obtained from fresh PBMCs the same day as collection using a state-of-the-art Seahorse XFe96 Analyzer (Seahorse Bioscience, Inc., North Billerica, MA). The Seahorse analyzer measures oxygen consumption rate (OCR) in real-time in a 96-well plate in a wide range of intact living cell types [43, 44]. The assay measures several key parameters: Adenosine triphosphate (ATP) Linked Respiration (ALR), Proton Leak Respiration (PLR), Maximal Respiratory Capacity (MRC), a parameter that is sensitive to deficits in mitochondrial biogenesis, mtDNA damage and/or inhibition of ETC function, and Reserve Capacity (RC), a parameter which determines the threshold at which bioenergetic dysfunction occurs [45]. In addition, mitochondrial function in peripheral blood cells has been proposed as a biomarker of bioenergetic health for a wide range of conditions [46], including adiposity [47], porphyria [48], postoperative cardiac surgery [49], and diabetic neuropathy [50]. Mitochondrial function in PBMCs of children with ASD has been measured using the Seahorse XFe96 in siblings with ASD and genetic abnormalities [51], in children being evaluated for immune abnormalities to demonstrate the correlation between cytokine profiles and mitochondrial function in children with ASD [52] and in a cohort of children with ASD with and without NDR [25].

PBMCs were placed in assay media (unbuffered RPMI supplemented with 1 mM pyruvate, 2 mM glutamate and 25 mM glucose) that was warmed to 37 °C and pH adjusted to 7.4 prior to cell suspension. XFe96 plates (Seahorse Bioscience, Billerica, MA) were prepared by adding 25 μL of 50 μg/mL Poly-D-lysine (EMD Millipore, Billerica, MA) for 2 h, washing with 250 μL sterile water and drying in a laminar flow hood overnight prior to seeding with 4 × 10^5^ viable PBMCs per well. After seeding, the plates were spun with slow acceleration (4 on a scale of 9) to a maximum of 100 g for 2 min and then allowed to stop with zero braking (Eppendorf Model 5810 R Centrifuge). The plate orientation was reversed, and the plate was spun again to 100 g in the same fashion. Prior to Seahorse assay, XFe96 wells were visualized using an inverted microscope to ensure PBMCs were evenly distributed in a single layer and viability of the cells was confirmed by trypan blue exclusion.

For each experimental condition, four replicate samples were measured simultaneously to improve assay reliability. Runs with clear measurement probe failure, reagent injection failures or nonphysiology measurements (ALR or PLR <−1 pmol/min) were eliminated. Results from each sample run were included in the analysis to improve the accuracy of the analysis and fully represent the variation in the assay measurements. This resulted in 285 data points for those participants with NDR and 261 data points for participants without NDR.

**Fig. 2:**
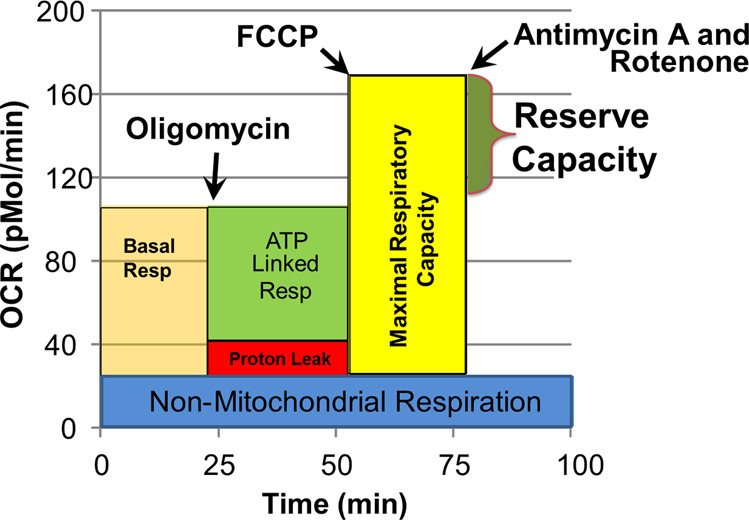
Depiction of the Seahorse assay and derived mitochondrial respiratory parameters. Oxygen consumption rate (OCR) is measured to determine mitochondrial activity. Three OCRs are measured over an 18 min period to determine mitochondrial activity for each segment of the assay. Regents are added to determine parameters of mitochondrial activity. Basal Respiration is the difference between baseline OCR and non-mitochondrial OCR. Oligomycin, which is a complex V inhibitor, is added to determine the portion of Basal Respiration that is ATP-Linked Respiration and Proton-Leak Respiration. Carbonyl cyanide-p-trifluoromethoxyphenyl-hydrazon (FCCP), a protonophore, is added to collapse the inner membrane gradient, driving the mitochondria to respire at its maximal rate: this determines Maximal Respiratory Capacity. Antimycin A and Rotenone, complex I and III inhibitors, stop mitochondrial respiration in order to determine the non-mitochondrial respiration. Reserve Capacity is the difference between Basal Respiration and Maximal Respiratory Capacity.

As seen in Fig. 2, the assay is a 4-step process which monitors OCR in response to various reagents that activate or inhibit ETC complexes.

- Baseline OCR: Baseline OCR is measured before introducing any reagents.
- OCR after oligomycin: Oligomycin, a complex V inhibitor, shuts down the production of ATP so the OCR related to ATP production can be determined.
- Maximal OCR: Carbonyl cyanide-p-trifluoromethoxyphenyl-hydrazone (FCCP) is used to collapse the mitochondrial inner membrane gradient, inducing the mitochondria to function at its maximum extent possible.
- Non-Mitochondrial OCR: Antimycin A and Rotenone are added to shut down ETC complex activity to measure OCR from non-ETC processes.

The following measures are derived from the above measurements:

- ATP-Linked respiration (ALR): The portion of basal OCR that is attributed to ATP production.
  ATP-Linked respiration = Baseline OCR - OCR after oligomycin
- Proton-Leak Respiration (PLR): The amount of basal OCR that is associated with protons leaking through the inner mitochondrial membrane in order to control oxidative stress.
  Proton-Leak respiration = OCR after oligomycin - Non-Mitochondrial OCR
- Maximal Respiratory Capacity (MRC): The maximum respiratory rate of the ETC.
  Maximal Respiratory Capacity = Maximal OCR - Non-Mitochondrial OCR
- Reserve Capacity (RC): The amount of extra ATP that can be produced by oxidative phosphorylation when there is a sudden increase in energy demand. This parameter is an index of mitochondrial health; when it becomes negative, the mitochondrion is unhealthy leading toward apoptosis.
  Reserve Capacity = Maximal OCR - Baseline OCR

To examine reliability of the Seahorse respiratory measurement replicates, the single measure intraclass correlation coefficient was calculated for each respiratory measure using the second model absolute agreement as estimated by a two-way mixed effects model. Intraclass correlation coefficient (95% confidence interval) were: ALR = 0.939 (0.921,0.955); PLR = 0.721 (0.647, 0.788); MRC = 0.963 (0.951, 0.972); RC = 0.952 (0.938, 0.964).

### Redox biomarkers

High Performance Liquid Chromatography with electrochemical detection was used to measure intracellular reduced glutathione (iGSH) and oxidized glutathione (iGSSG) from frozen PBMC within 2 weeks of collection [53, 54]. These values were used in the SEM analysis.

### Statistical analysis

Regression and mediation analyses were performed using SAS 9.4 (SAS Institute Inc., Cary, NC). Graphs were produced using Excel version 14.0 (Microsoft Corp, Redmond, WA). Variables were normally distributed and met the assumption of the analysis and variation was similar across groups studied. A mixed-model linear regression was used to account for both within-subject variation from repeated mitochondrial measurements on the same individual and the between-subject variation from variables such as NDR and air pollution variable (PM_2.5_) using proc “glimmix” in SAS. In general, an *α* = 0.05 was used. However, for the main analyses investigating the relationship between mitochondrial function and air pollution (PM_2.5_), the Bonferroni correction (0.05/4 = 0.0125) was used because of the multiple (four) respiratory measures which were repeatedly tested with each measure of air pollution. For analyses without within-subject variation, general linear models were performed using proc ‘genmod’ in SAS. In these linear models, covariates of sex and age were added but removed from the final models if they were not significant. They are reported where significant. In models which used behavior measures (i.e., SRS, ABC) as dependent variables, the VABS Adaptive Behavioral Composite was used as a covariate since these behavioral measures depend on neurodevelopment. VABS is an important predictor of functional abilities in those with ASD [55, 56] and children at high-risk for ASD [57, 58] and has been shown to be an important covariate to include when studying ABC and SRS in our previous studies [40, 41].

To confirm and complement the mixed-model analysis, bivariate Pearson correlation coefficient *r* along with the *p* value for the r are presented in the scatterplots. *r* values are a standardized measures of relationship strength with the following conventions: *r* of 0.1–0.3 is a small effect, *r* of 0.3–0.5 is a medium effect and *r* > = 0.50 is a large effect [59]. For simple group comparisons the Cohen *d’* effect size was reported for all significant differences. Power analysis was calculated by G*Power version 3.1.9.4 (Kiel, Germany) [60, 61]. For a linear regression analysis of the relationship between air pollution and mitochondrial function mediated by another variable, a power analysis assuming a medium effect size *f*^2^ = 0.15 and *N* = 96 finds a power of 89% for *α* = 0.05 and 75% for *α* = 0.0125. The power drops considerably for a small effect size *f*^2^ = 0.05, with a power of 40% for *α* = 0.05 and of 21% for *α* = 0.0125. For linear regressions between two variables (for example air pollution and a specific mitochondrial measures) without a mediating variable, a power analysis assuming a medium effect size *f*^2^ = 0.15 and *N* = 96 finds a power of 96% for *α* = 0.05 and 89% for *α* = 0.0125. The power drops considerably for a small effect size *f*^2^ = 0.05, with a power of 58% for *α* = 0.05 and of 37% for *α* = 0.0125.

Mediation analysis determines whether a mediator variable “M” accounts for an indirect effect of a treatment variable “T” on an outcome variable “Y”. In this manuscript mediation analysis was used to determine whether mitochondrial respiration (“M”) accounted for some of the effect of air pollution (“T”) on behavior or neurodevelopment (“Y”) [62]. Mediation analysis was performed using proc CAUSALMED in SAS which follows the definitions and methods of VanderWeele and Vansteelandt [63]. Covariates were used as indicated by previous studies [40, 41]; specifically VABS was used as a covariate for analyses that contained SRS and ABC scores as outcomes. Mediation analysis was considered for those relationships where mitochondrial function was found to effect neurodevelopment and behavior as determined in separate linear regression analysis presented in supplementary analysis section. Specifically, only PLR was found to influence ABC scores, so for the ABC analysis, only the PLR variable was examined.

SEM was implemented by the lavaan (v0.3–6) on RStudio (v3.5.1) to better understand the interrelationships between air pollution, mitochondrial function, redox metabolism and behavior and neurodevelopment. Variables were standardized within the respective dataset. Relevant models were built and compared. In general, separate variables were used for (1) environmental exposure, (2) mitochondrial energy production, (3) mitochondrial oxidative stress control, (4) redox metabolism (both iGSH and iGSSG), (5) cognitive neurodevelopment, (6) behavior and (7) age of NDR in the models for those with NDR. These models did introduce measures of redox metabolism since it is so important in regards to mitochondrial function in ASD [64], especially with respect to NDR [25, 26, 30, 31], and is a prominent physiological abnormality found in ASD [53, 65, 66]. Since ASD behavior depended on neurodevelopment in the regression models above as well in our previous studies [40, 41], a relationship between neurodevelopment and behavior was considered in the models and was found to be significant in every model. Models were fit to minimize the Chi Square Model Fit Test Statistic as well as meet or exceed the standard model fit statistics. In general, only significant (*p* < 0.05) coefficients were kept in the model. Once a model for each group was developed, the parameters were calculated with confirmatory factor analysis.

We developed separate SEM models for participants with and without NDR because previous studies suggest that mitochondrial function in ASD, especially in relation to handling oxidative stress, is different depending on the history of NDR [25, 26, 30, 31]. Thus, this potential difference in the relationship between mitochondrial function and other variables can complicate the fitting of the SEM model if data from both those with and without NDR are included in the same model for several reasons. First, developing a SEM model requires fitting an optimal model which differs from the null model in which all variables are connected. If some of the connections are valid for a subset of the data and other are valid for another subset, then it will be difficult to find an optimal model. Second, it is possible that a model could be built with interactions to account for the different relationship for those with and without NDR. However, this would not only add model terms to represent different connections between variables for those with and without NDR but we would need to add interaction terms to represent NDR status. Thus, the number of variables in a model which combines both NDR groups would be considerably more (at least twice the number of variables) than either of the models which considered the NDR groups separately. Thus, the ability to estimate the true models, especially the values of the model coefficients, would be reduced by the substantial increase in the variables in a model that contained both NDR groups. Thus, we developed separate SEM models for those with and without NDR. Coefficients of the SEM models were compared within each NDR group with respect to the variable used (e.g., ^ave^PM_2.5_ vs ^max^PM_2.5_) using *t* tests. Coefficients of SEM models across NDR groups were compared by calculating the difference between the *z* values of the coefficients and determining the *p* value based on a z-distribution.

The number of data points (with NDR = 285; without NDR = 261) in each SEM conforms to the guidelines regarding the minimum sample size of 200, 10 observations per estimated parameter and 10 cases per variable as discussed in recent studies using Monte Carlo data simulation techniques to evaluate sample size requirements for SEM [67]. The fit indices recommended by Hu and Bentler [68] were used to minimize Type I and II errors. Recommendations included using a combination of absolute, relative, and noncentrality-based indices. Thus, model fit was assessed using the Comparative Fit Index, Root Mean Square Error of Approximation and Standardized Root Mean Square Residual as calculated by lavaan ‘cfa’ function, and Gamma Hat as calculated by the “moreFitIndices” function from semTools (v0.5–2) in RStudio (v3.5.1). All indices are reported in the Supplementary tables and are within the recommend cutoffs. In addition, to confirm that the models were adequately powered and no variables were misspecified, the “miPowerFit” function in semTools was implemented with parameters suggested by Saris and Satorra [69]. Specifically, the following parameters values were specified in ‘miPowerFit’: standardized factor loading to detect = 0.4, error correlation to detect = 0.1, standardized regression coefficients to detect = 0.1, standardized intercept to detect = 0.2, and confidence interval of expected parameter change = 0.9. Since the standardized intercept to detect is similar to the Cohen *d’*, the parameters are set to test the power of detecting a small effect size. None of the variables used in the model were misspecified and only one set of models (those that used the SRS with ^max^PM_2.5_ for participants with a history of NDR) did not have optimal values for all fit statistics, although even these nonoptimal models were close to meeting these standards.

## Results

Here we discuss the main hypothesized relationships: the relationship between air pollution and mitochondrial function and whether such a relationship accounts for any effect of air pollution on neurodevelopment and/or behavior. To assist in readability, analysis looking at other relationships between the variable studied are presented in the Supplementary results.

To provide a perspective of the values of the outcome measures in ASD, overall comparisons between those with ASD with and without NDR and TD controls with respect to mitochondrial function and PM_2.5_ is given in Supplementary Table S2. Overall mitochondrial respiration was not different between those with ASD and controls although those with ASD and a history of NDR did demonstrate significantly higher PLR as compared to those with ASD without a history of NDR. Overall, individuals with ASD demonstrated higher PM_2.5_ concentrations as compared to TD controls, consistent with previous studies [42] but this difference was only borderline significant most likely due to the small TD sample size.

### Prenatal air pollution is related to long-term variations in mitochondrial respiration

**Fig. 3:**
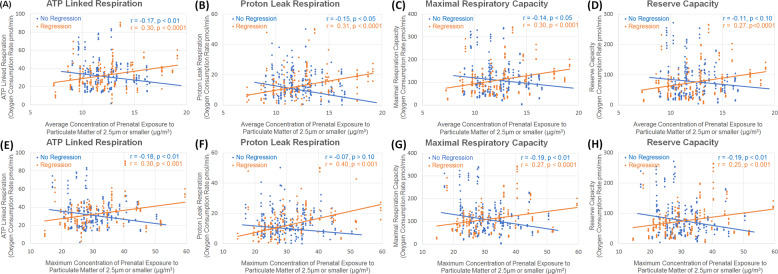
The relationship between four measures of mitochondrial respiration and prenatal air pollution exposure for children with autism spectrum disorder with (orange line/dots) and without (blue line/dot) a history of neurodevelopmental regression. The two different groups of children demonstrate opposite relationships between mitochondrial respiration and prenatal exposure to air pollution such that for those with a history of neurodevelopmental regression exposure to higher prenatal concentrations of air pollution is associated with long-term higher mitochondrial respiratory rates while for those without a history of neurodevelopmental regression exposure to higher prenatal concentrations of air pollution is associated with lower long-term mitochondrial respiratory rates.

As hypothesized, a significant relationship was found between mitochondrial respiration and both ^max^PM_2.5_ and ^ave^PM_2.5_. This relationship was dependent on the history of NDR as demonstrated by statistically signficant interactions between NDR and respiratory parameters (ALR, PLR, MRC, RC). Overall, for those with a history of NDR, higher prenatal ^max^PM_2.5_ or ^ave^PM_2.5_ exposure was related to higher mitochondrial respiration while for those without a history of NDR, higher prenatal ^max^PM_2.5_ or ^ave^PM_2.5_ exposure was related to lower mitochondrial respiration (See Fig. 3). Follow-up regression analysis for each NDR group demonstrated signficance for all relationships except for the relationship between PLR and ^max^PM_2.5_ and ^ave^PM_2.5_ and RC for those without NDR (See Supplementary Material).

Correlations were stronger for the relationship between mitochondrial respiration and PM_2.5_ for those with a history of NDR (*r* = 0.25–0.40; small-to-medium effect sizes) than those individuals without a history of NDR (*r* = −0.07 to −0.19; small effect sizes). The correlation coefficients were significant for all relationships between PM_2.5_ and mitochondrial respiration for children with a history of NDR while all but two relationships between PM_2.5_ and mitochondrial respiration were significant for those children without a history of NDR (See Fig. 3).

### The effect of prenatal air pollution exposure on neurodevelopment and behavior is partially driven by its effect on mitochondria respiration

To investigate whether the effect of air pollution on neurodevelopment (VABS) and behavior (SRS, ABC) is the result of an indirect effect through the mitochondria, a mediation analysis was conducted. The mediation analysis also allows the estimation of the direct effect of air pollution on behavior and neurodevelopment while accounting for any effect of the mitochondria on behavior and neurodevelopment.

**Fig. 4:**
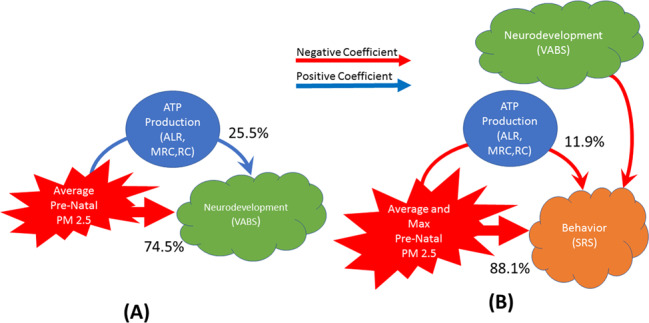
The direct and indirect effects of air pollution on behavior and neurodevelopment. The direct and indirect effect of air pollution on (**a**) neurodevelopment (as measured by the Vineland Adaptive Behavior Scale) and (**b**) social behavior (as measured by the Social Responsiveness Scale). The indirect effect of air pollution is measured as the percentage of the total effect accounted for by mitochondrial respiration parameters which is shown next to the arrows. Note that neurodevelopment (VABS) was a covariate in the analysis that examined the effect of air pollution on behavior. The color of the arrows indicates whether the coefficients in the analysis are positive (blue) or negative (red).

^ave^PM_2.5_ had both a significant direct and indirect effects on VABS through mitochondrial respiration associated with ATP production (ALR, MRC, RC). The direct effect of ^ave^PM_2.5_ on VABS was negative suggesting that higher prenatal ^ave^PM_2.5_ exposure was associated with lower neurodevelopment. However, the indirect effect ^ave^PM_2.5_ on VABS through mitochondrial respiration associated with ATP production (ALR, MRC, RC) was positive, suggesting a higher prenatal ^ave^PM_2.5_ exposure was associated with a higher VABS score through an increase in mitochondrial respiration. Thus, the direct and indirect effects of ^ave^PM_2.5_ on neurodevelopment were in opposite directions. The indirect effect accounted for 26%, 26% and 24% [Average 25.5% (2.1%)] for ALR, MRC, RC, respectively, of the total effect of ^ave^PM_2.5_ on VABS. PLR did not account for any indirect effect of ^ave^PM_2.5_ on VABS (Fig. 4a). ^max^PM_2.5_ had both a significant direct and indirect effect on VABS only for PLR where the indirect effect of PLR was found to account for 22% of the total effect of ^max^PM_2.5_ on VABS (See Supplementary Table S3).

Both ^ave^PM_2.5_ and ^max^PM_2.5_ had significant direct and indirect effects on SRS for mitochondrial respiration associated with ATP production. Mitochondrial respiration associated with ATP production accounted for 10.2% (SD = 1.5%) of the total ^ave^PM_2.5_ effect (ALR = 8.5%, MRC = 11.4%, RC = 10.7%) and 13.7% (SD = 0.8%) of the total ^max^PM_2.5_ effect (ALR = 12.9%, MRC = 14.5%, RC = 13.8%) on SRS (Fig. 4b). Both the direct and indirect effects of PM_2.5_ had negative coefficients. Thus, higher PM_2.5_ was associated with lower SRS scores through the direct effect of prenatal PM_2.5_ exposure on the SRS scores and through the indirect effect of prenatal PM_2.5_ exposure on SRS scores through its effect on respiratory parameters associated with ATP production. For ^max^PM_2.5_ PLR accounted for 14.5% of the total effect of ^max^PM_2.5_ on SRS with the influence of both the direct and indirect effect the same as the respiratory parameters associated with ATP production (See Supplementary Table S4).

Since only PLR was found to influence ABC, a mediation analysis was performed but PLR was not found to significantly mediate any effects of air pollution on ABC (Supplementary Table S5).

### Understanding the complex relationships: structural equation modeling (SEM) analysis

In order to gain a better understanding of the direct and indirect effects of air pollution on neurodevelopment and behavior as well as the role of the mitochondria and other factors, SEMs were constructed from variables representing air pollution, mitochondria and redox metabolism as well as neurodevelopment and behavioral symptoms using the guidance of the previous analyses. SEMs allowed the consideration of the simultaneous complex interactions of several factors which could be modeled in the linear models. For example, the use of SEMs allowed consideration of whether the effect of mitochondrial function on behavior was through neurodevelopment or whether it had a separate distinct effect on behavior. SEMs also allowed consideration of the influence of redox metabolism on mitochondria and neurodevelopment and behavioral symptoms, and the separate influence of mitochondrial respiratory parameters associated with energy production (ALR, MRC, RC) from the mitochondrial respiratory parameter associated with control of oxidative stress (PLR).

**Fig. 5:**
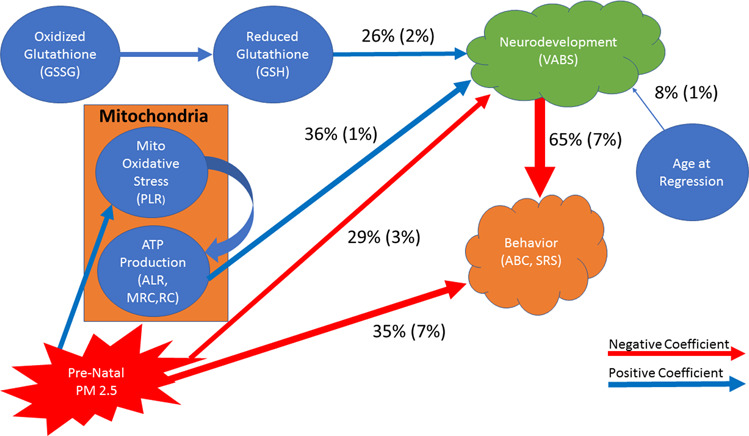
Summary depiction of the structural equation model for children with autism spectrum disorder and a history of neurodevelopmental regression (Traditional Diagram given as Supplementary Fig. S2). The model verified both the direct and indirect influences of prenatal air pollution (PM_2.5_) exposure through the mitochondria and found that the mitochondrial has slightly more influence on neurodevelopment than PM_2.5_. For individuals with neurodevelopmental regression PM_2.5_ primarily influences mitochondrial mechanisms associated with the control of oxidative stress as compared to mitochondrial respiration associated with energy production. Blue arrows depict positive coefficients and red arrows depict negative coefficients. The thickness of the arrows is proportional to the relative size of the effect compared to other effects on the target variable.

**Fig. 6:**
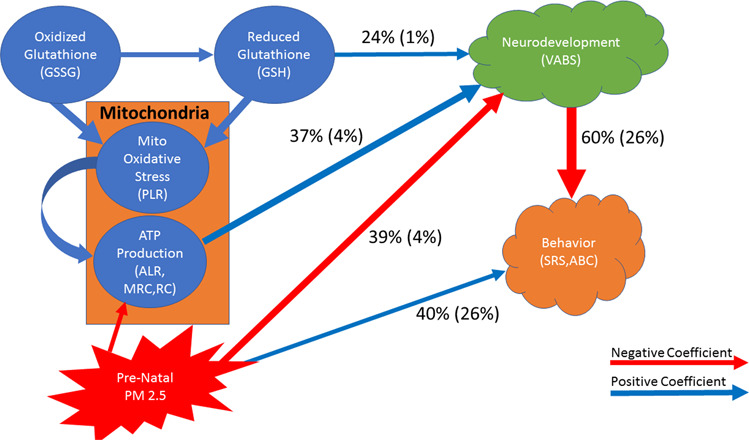
Summary depiction of the structural equation model for children with autism spectrum disorder without neurodevelopmental regression (Traditional Diagram given as Supplementary Fig. S3). The model verified both the direct and indirect influences of prenatal exposure to air pollution (PM2.5) through the mitochondria which is different than children with a history of neurodevelopmental regression. The effect of PM_2.5_ on behavior is opposite to that for children with neurodevelopmental regression. Lastly, the interaction of glutathione metabolism and mitochondrial function different than those ASD children with a history of neurodevelopmental regression. Blue arrows depict positive coefficients and red arrows depict negative coefficients. The thickness of the arrows proportional to the relative size of the effect compared to other effects on the target variable.

Neurodevelopment (as measured by the VABS) and behavioral symptoms (as measured by the ABC and SRS total score) were considered as separate factors in the model. For model development, various theoretical pathways were tested such as PM_2.5_ influencing mitochondrial, redox physiology, neurodevelopment and behavioral symptoms; mitochondrial physiology influencing redox, neurodevelopment and behavioral symptoms; and redox influencing mitochondrial physiology as well as neurodevelopment and behavioral symptoms. Using the different variations of behavioral variables (ABC, SRS), PM_2.5_ air pollution variables (average, maximum) and mitochondrial variables (ALR, PLR, MRC, RC), it was found that separate models were needed for participants with and without NDR because of a different relationship between air pollution, mitochondrial respiration, and redox metabolism. The estimated coefficient values along with their *p* values and fit statistics for the models are given in Supplementary Tables S6 and S7. General depictions of the models are given in Figs. 5 and 6. For the neurodevelopment and behavioral variables, average (standard deviation) percentage influence of each factor on the total symptoms is outlined in the diagram. Formal SEM diagrams are given in the Supplementary Results Figs. S2 and S3. The average effects in the models are discussed followed by highlighting the significant differences between the different variations of the models and the differences in relationships between variables in the SEMs for those with and without a history of NDR.

For participants with NDR, PM_2.5_ had an indirect effect on neurodevelopment through mitochondrial respiration, but the relationship was more complex than previously considered in the mediation analysis. PM_2.5_ had an indirect effect on ATP production (ALR, MRC, RC) by positively effecting PLR which in turn influenced mitochondrial parameters associated with ATP production rather than PM_2.5_ influencing parameters associated with ATP production directly (Fig. 5).

Overall, for participants with a history of NDR, neurodevelopment was influenced by four factors. Mitochondrial respiration, PM_2.5_, GSH concentration and the age of the NDR, which accounted for 36% (SD 1%), 29% (SD 3%), 26% (SD 2%) and 8% (SD 1%) of the overall effect on neurodevelopment, respectively (Fig. 5). Thus, overall mitochondrial function has slightly more influence on neurodevelopment as compared to air pollution, although a portion of the effect of the mitochondria on neurodevelopment was driven indirectly by the influence of air pollution. The relative magnitude of these four factors were influenced by the PM_2.5_ exposure variable used in the model. Specifically, when ^ave^PM_2.5_ was used in the model, air pollution was found to have a greater influence on neurodevelopment [33% (SD 0.5%)] as compared to when ^max^PM_2.5_ exposure was used in the model [26% (SD 1%); *t*(5) = 23.80, *p* < 0.0001]. In models that included ^ave^PM_2.5_, because of the increased influence of ^ave^PM_2.5_ exposure, neurodevelopment was less influenced by both age of regression [7% (SD 0.2%) vs 9% (SD 0.1%); *t*(5) = 26.09, *p* < 0.0001] and GSH concentration [25% (SD 0.7%) vs 28% (SD 0.6%); *t*(5) = 15.00, *p* < 0.001] than models which included ^max^PM_2.5_.

Both neurodevelopment and PM_2.5_ exposure had a negative effect on ABC or SRS scores, which translates to better behavior. Neurodevelopment accounted for most of the effect on behavior [65% (SD 7%)] while PM_2.5_ exposure accounted for less of the effect [35% (SD 7%)]. These effects were not different across parameters of ATP production but were affected by the behavioral instrument and the PM_2.5_ exposure variables. Neurodevelopment had a significantly greater influence on the ABC score [72% (SD 4%)] as compared to the SRS score [59% (SD 1%); *t*(5) = 8.82, *p* < 0.001] and had a greater influence on behavior in general when the ^max^PM_2.5_ exposure [68% (SD 9%)] was used in the model as compared to when ^ave^PM_2.5_ exposure was used in the model [63% (SD 5%); *t*(5)=3.37, *p* < 0.05].

For participants without a history of NDR, PM_2.5_ had an indirect effect on neurodevelopment through mitochondrial respiration. PM_2.5_ negatively influenced mitochondrial parameters associated with ATP production (ALR, MRC, RC). Air pollution was found to have a direct effect on mitochondrial parameters associated with ATP production (Fig. 6). Neurodevelopment was influenced by three factors. Mitochondrial respiration, PM_2.5_ and GSH concentration accounted for 37% (SD 4%), 39% (SD 4%) and 24% (SD 1%) of the overall effect on neurodevelopment, respectively (Fig. 3b). The magnitude of influence of these effects depended on the PM_2.5_ exposure variable used in the model. When the ^max^PM_2.5_ variable was used, air pollution was found to have a greater influence on neurodevelopment [43% (SD 0.9%)] as compared to when the ^ave^PM_2.5_ variable was used [35% (SD 1%); *t*(5)=30.36, *p* < 0.0001]. Because of the increased influence of ^max^PM_2.5_ exposure, neurodevelopment was less influenced by both mitochondrial respiration [34% (SD 1.5%) vs 41% (SD 1%); *t*(5)=36.86, *p* < 0.0001] and GSH concentration [23% (SD 0.7%) vs 24% (SD 0.8%); *t*(5)=11.82, *p* < 0.001] in models that use ^max^PM_2.5_ as compared to the models that used ^ave^PM_2.5_.

Similar to those with NDR, behavior was influenced by both neurodevelopment and PM_2.5_ exposure for those without a history of NDR. However, the direction of the effect of PM_2.5_ on behavior was different for those without NDR; better neurodevelopment resulted in lower (better) behavioral scores while higher PM_2.5_ exposure was associated with higher (worse) behavioral scores. Overall, neurodevelopment accounted for most of the effect on behavior [60% (SD 26%)], while PM_2.5_ exposure accounted for a smaller portion [40% (SD 24%)]. These depended on the behavior symptoms instrument used. Neurodevelopment had a significantly greater influence on the SRS score [84% (SD 9%)] as compared to the ABC score [36% (SD 1%); *t*(5) = 12.63, *p* < 0.001] while PM_2.5_ exposure had a greater influence on ABC score [64% (SD 1%)] as compared to SRS score [16% (SD 10%)]. In fact, the effect for PM_2.5_ exposure was not significant for a direct effect on SRS scores.

We also compared the common parameters between the NDR and non-NDR SEMs (See Supplementary Table S8). The effect of PLR on MRC [0.64 (0.11) vs 0.42 (0.14)] and RC [0.58 (0.11) vs 0.25 (0.15)] was significantly greater for individuals with a history of NDR as compared to those without a history of NDR. Neurodevelopment had a significantly greater influence on the ABC for individuals with a history of NDR as compared to those without a history of NDR [−0.39 (0.13) vs −0.21 (0.13)]. The effect of PM_2.5_ exposure on behavior symptoms (SRS and ABC) was significantly different because it had an opposite effect for individuals with NDR as compared to those without NDR. In addition, although the effect of PM_2.5_ on neurodevelopment was lower for the models for individuals with a history of NDR this difference in influences was only significant for ^max^PM_2.5_ [−0.15 (0.16) vs −0.40 (0.24)].

## Discussion

Prenatal exposure to air pollution is a known risk factor for developing ASD [7] and, separately, has been shown to disrupt mitochondrial respiration [13]. This study aimed to determine if air pollution has a long-term effect on mitochondrial respiration and whether this effect may indirectly mediate the effect of air pollution exposure on neurodevelopment and behavior. We hypothesized that exposure to air pollution during the prenatal period was associated with prolonged changes in mitochondrial respiration, a key physiological system known to be disrupted in children with ASD and influenced by environmental factors. Overall, we found that prenatal air pollution exposure is associated with long-term changes in mitochondrial function and that mitochondria may indirectly mediate some of the effect of air pollution on neurodevelopment and behavior. Furthermore, many of these relationships may be different depending on whether the child has a history of NDR.

We examined two measures of prenatal air pollution: ^ave^PM_2.5_ and ^max^PM_2.5_. Both measures of air pollution were associated with long-term changes in mitochondrial function. Interestingly, as hypothesized, the relationship between prenatal air pollution exposure and mitochondrial respiration depended on the history of NDR. Those with a history of NDR demonstrated a positive relationship between prenatal air pollution exposure and mitochondrial respiration involved in ATP production (ALR, MRC, RC) and control of oxidative stress (PLR), such that mitochondrial respiration increased with higher prenatal exposures to air pollution with small-to-medium sized effects. Whereas those without a history of NDR demonstrated a negative relationship such that mitochondrial respiration decreased with greater prenatal exposure to air pollution with small sized effects.

Given the fact that air pollution could affect mitochondrial respiration, and that air pollution may influenced neurodevelopment and ASD symptoms, we conducted analyses to examine how much of the effect of air pollution on neurodevelopment and behavior was indirectly influenced by the effect of air pollution on mitochondrial respiration. Using mediation analysis, we found that about 25% of the effect of air pollution on neurodevelopment was mediated by mitochondrial respiration associated with ATP production. SEM analysis confirmed an indirect influence of air pollution on neurodevelopment for both those with and without NDR and estimated that neurodevelopment was influenced about equally by air pollution, mitochondrial function, and redox metabolism. The mediation analysis found that about 12% of the effect of air pollution on behavior was mediated through mitochondrial function. This was clarified in the SEMs which suggested that the indirect effect of air pollution on behavior through mitochondrial function was probably mediated by second and third level indirect relationships through neurodevelopment. Thus, while mitochondrial function did appear to have some association with behavioral symptoms in initial analyses, the SEMs suggested that these physiological factors indirectly influenced ASD symptoms through their influence on neurodevelopment.

### The effect of air pollution on mitochondrial function depends on neurodevelopmental regression

The influence of PM_2.5_ exposure on mitochondrial respiration was different for those with and without NDR. Higher levels of PM_2.5_ exposure during gestation was related to lower mitochondrial respiration in childhood for those without a history of NDR. In addition, the SEMs suggested that this was a direct effect of mitochondrial respiration associated with ATP production rather than mitochondrial respiration associated with control of oxidative stress (i.e., PLR). In contrast, for individuals who had a history of NDR, higher levels of PM_2.5_ exposure during gestation was related to higher mitochondrial respiratory. The SEMs suggested that the effect of PM_2.5_ exposure on ATP production by the mitochondria was indirect by influencing the mitochondrial mechanism for controlling oxidative stress (i.e., PLR). The differences in the effect of PM_2.5_ exposure on long-term mitochondrial function for those with and without NDR suggest that underlying genetic and epigenetic factors which program individuals to respond differently to physiological and environmental stressors play a role in the response of the mitochondria to air pollution.

### Parallels with laboratory models of mitochondrial dysfunction in ASD

The positive association between air pollution and mitochondrial respiration for the individuals with NDR parallels our in vitro cell model of mitochondrial dysfunction in ASD. Specifically, about one-third of LCLs derived from children with ASD demonstrate high respiratory rates, ~200% of controls, for parameters associated with ATP production; this subset is referred to as AD-A LCLs. AD-A LCLs are more sensitive to acute exposure to reactive oxygen species (ROS) such that respiratory rates drop precipitously with acute increases in ROS. We have suggested that this may represent a model of mitochondrial associated NDR as mitochondrial function is lost with a mild increase in ROS in the AD-A LCL model and NDR is known to be associated with triggers that increase physiological stressor which can increase ROS. In fact, a recent study examining mitochondrial function in PBMCs in children with ASD in a manner similar to the current study found that those with a history of NDR demonstrated elevated respiratory rates, similar to our LCL model, as compared to those without a history of NDR [25].

AD-A LCLs show the same elevated respiratory rates at baseline in repeated experiments and respond differently to environmental agents associated with ASD, including trichloroacetaldehyde hydrate [70] and ethylmercury, [32] and enteric short chain fatty acids propionate [40] and butyrate, [28] as compared to ASD LCLs which do not demonstrate these high respiratory rates at baseline (called AD-N LCLs). These changes may represent mitoplasticity in AD-A LCLs in an attempt to adapt to changes in the microenvironment. [22] We recently demonstrated that elevated respiratory rates can be induced in LCLs with prolonged exposure (96 hr) to mild ROS, a microenvironment that simulates the effect of toxicants on the mitochondria [22]. As air pollution can increase ROS [13, 71, 72], it is possible that air pollution can cause a similar oxidized microenvironment, resulting in long-term changes in mitochondrial respiration. This notion would be consistent with recent studies which demonstrate that air pollution, particularly PM_2.5_, can alter mtDNA, presumably though oxidative damage [10–12], and that prenatal air pollution exposure increases cord blood concentrations of a mitochondrial-derived peptides that are associated with long-term mitochondrial metabolism alterations [13].

The important and separate influence of PLR from parameters of mitochondrial respiration involved in ATP production, particularly its role in mediating the effects of air pollution on mitochondrial function in children with NDR, parallels previous studies on mitochondrial dysfunction in ASD. PLR is regulated, in part, by uncoupling proteins which are an integral part of the inner mitochondrial membrane. Three previous studies that found an increase in uncoupling protein (UCP2) expression [26, 73] and protein [31] in LCLs derived from children with ASD, particularly the AD-A LCLs [31]. In addition, UCP2 expression was one of the genes that discriminated AD-A and AD-N LCLs using canonical discriminant analysis [22]. PLR is critical for regulating ROS at the at the inner mitochondrial membrane where the ETC functions. This is consistent with the notion that previous exposure to a physiological stressor, such as air pollution, may alter long-term mitochondrial function by changing the mitochondria’s response to ROS regulation.

Elevated mitochondrial respiration, similar to what is seen in our LCL model, has been reported in other studies of mitochondrial function related to ASD. Elevations in ETC activity has been documented in multiple tissues in children with ASD [19, 29, 74–77]. Two recent studies have also verified this pattern of elevated respiratory rates. Recently high-resolution respirometry verified elevated respiratory rates in ASD LCLs [78], particularly the activity of Complex IV. This complements the results of our case-series of Complex IV overactivity in muscle biopsies from patients with ASD [75]. Recently Percorelli et al. [79] documented elevated respiratory rates with associated changed in mitochondrial morphology in fibroblasts from individuals with ASD. Interestingly, both Percorelli et al. [79] and our group [22] found changes in the expression of genes important for maintaining appropriate mitochondrial dynamic and repair which affect morphology. Our recent LCL study demonstrated that these changes may be different for subsets of individuals with ASD based on whether there is underlying mitochondrial dysfunction. Furthermore, elevated ETC activity is found in brain tissue from the maternal immune activation mouse, a prenatal environmentally induced model of ASD [80], further suggesting that prenatal environmental stressors may alter the physiology of the mitochondrial in ASD. Thus, these novel changes in mitochondrial respiration associated with ASD may be a prime example of environmentally induced long-term changes in mitochondrial function.

### Elements of air pollution can directly affect mitochondrial metabolism

Air pollution contain both naturally sourced crustal metals: Ca, Mg, Na, K, Al, Si, Fe, Ti, Mn, and anthropogenically sourced metals: V, Cr, Ni, Zn, Cd, Pb, Cu [81, 82], many of which are known to be toxic to the mitochondria. As an example, Pb exposure correlates with PM_2.5_ exposure [83] and adversely affects the immune and nervous systems, including behavioral problems, learning deficits, intellectual disability and ASD [84]. In animal models, Pb exposure results in mitochondrial ultrastructure damage, and/or dysfunction in neuronal tissue [85–88], and depletes mitochondrial GSH in brain [89]. Pb also results in oxidative stress by depleting GSH [86, 89], decreasing superoxide dismutase activity [86, 89], and increasing malondialdehyde [89], ROS production [85], and catalase expression [89]. PM_2.5_ also contains other toxic and nutrient metals including cadmium, iron and nickel which can have detrimental effects on the mitochondria [6, 90]. Interestingly, we have recently found that prenatal exposure to nutrient metals Zinc and Manganese, both of which are contained in air pollution, modulate long-term mitochondrial function in children with ASD and NDR [91].

### Mitochondrial disease, neurodevelopmental regression and air pollution

This study found that the influence of prenatal air pollution on mitochondrial respiration was strongest for children with ASD with a history of NDR with the relationships demonstrating mostly medium effect sizes. This is of great interest as NDR from TD into ASD is associated with a mitochondrial disease diagnosis and it is not uncommonly triggered by an inflammatory event [15, 23, 24]. Data from our study aligns with the notion that children with NDR have vulnerable mitochondria and that this vulnerability was induced prenatally. In this manner, prenatal exposure to environmental toxicants or alterations in prenatal nutrients could predispose the mitochondria to be sensitive to post-natal physiological stressors [91], such as an infection or inflammatory event or other toxicant exposures. These post-natal stressors may act as a trigger, causing systemic metabolic decompensation, resulting in a precipitous loss of mitochondrial activity and deviation from the normal course of childhood development. The fact that air pollution influenced mitochondrial respiration through PLR which controls ROS at the ETC level, is consistent with this notion that ROS may be the trigger to disrupt mitochondrial respiration abruptly in those with NDR. Further research will be needed to determine the developmental course of this subset of children and what types of triggers (or lack of triggers) may have been associated with NDR events.

### Comparisons to other studies on air pollution in autism spectrum disorder

Several recent studies have demonstrated the association between air pollution and ASD [7] with some studies demonstrating factors which mitigate this association, including copy number increase [92], maternal folate intake [93], and MET receptor tyrosine kinase polymorpism [94]. However, despite identifying mitigating biological factors, these studies do not illuminate the physiological mechanisms which explain the effect of air pollution on brain function. A recent study showed that prenatal exposure to nonfreeway traffic-related air pollution was associated with mitochondrial-derived peptides in cord blood suggesting prenatal exposure to air pollution can result in long-term changes in mitochondrial physiology [13]. Furthermore, a recent animal study demonstrated that mitochondrial dysfunction during gestation resulted in abnormal white matter connectivity supporting the idea that mitochondrial function is important for prenatal brain development [14].

This study demonstrates the multiple direct and indirect effects of air pollution on neurodevelopment and ASD behaviors. These effects sometimes appear paradoxical as for those with a history of NDR, air pollution increases mitochondrial respiration which in turn is associated with better neurodevelopment as indexed by VABS scores. These multiple, sometimes opposing effects could explain some of the inconsistent and sometimes paradoxical findings in the literature such as air pollution being correlated with both an increase and decreased VABS scores [95]. Our study suggests that important factors such as NDR need to be taken into account to better understand the direction and magnitude of the indirect and direct effects of air pollution. NDR is a factor that has not been accounted for in previous studies that deserves attention in future studies.

### Study limitations

There are several limitations to this study. We have concentrated on the average and maximum PM_2.5_ exposures during the six months prior to the participant’s birth, although studies do suggest that there may be variations in susceptibility across trimesters, and even during the post-natal period. We also assume that the monitored pollution is a geospatially consistent snapshot across the entire zip code. The question regarding the exact components of PM_2.5_ exposure that could be responsible for causing disease needs to be addressed by future research. For example, toxic metals which are known to cause mitochondrial dysfunction are common PM_2.5_ components [81, 82, 96]. Furthermore, although the physiological changes studied (mitochondrial dysfunction and abnormal redox metabolism) are associated with ASD, the biological and clinical significance of these changes in relation to other neurodevelopmental disorders and diseases have not been well studied, so their significance is not entirely clear at this time. In addition, many maternal and pregnancy factors which have not been considered here are also a risk factor for ASD. Future studies will need to investigate how other prenatal factors influence long-term mitochondrial function in children with ASD. Lastly, the effects found in this study were small-to-medium size effects and the sample size of this study makes the power of the statistical analysis subpar for small effect sizes. Thus, many of the effects not found in this study cannot be ruled out, especially if they are small effects. As such, further studies with large sample sizes will be needed to rule out small effect of air pollution on the mitochondria, behavior and neurodevelopment.

## Conclusion

This study found that increased exposure to air pollution during gestation was associated with abnormalities in mitochondrial metabolism during childhood, although the exact relationship was dependent on whether the child had a history of NDR. Although preliminary, this study demonstrates that early life exposure to air pollution is associated with long-term alterations in mitochondrial physiology, possibly resulting in disease or increasing vulnerability to disease triggered by later life events. These data suggest that it is important to monitor the effects of air pollution and its specific components on the growing fetus as mitochondrial dysfunction has been implicated in a wide variety of brain-based diseases in which an environmental component is suspected, including psychiatric [97–100], neurodevelopmental [15], and neurodegenerative disorders [101]. Thus, the applications of these findings may be far reaching. Last, interventions to correct metabolic and mitochondrial abnormalities are under development, potential providing targeted treatments for preventing ASD and other diseases from developing.

## Supplementary information

Supplementary Material
