# Supplementary material for: Prenatal air pollution influences neurodevelopment and behavior in autism spectrum disorder by modulating mitochondrial physiology
Source: Mol Psychiatry. 2020 Sep 22;26(5):1561–77. doi: 10.1038/s41380-020-00885-2 (PMC8159748; doi:10.1038/s41380-020-00885-2)
Supplement: Supplementary file 1 — Supplementary Material [file 41380_2020_885_MOESM1_ESM.docx]

Supplemental Material

Molecular Psychiatry, submitted

Air pollution influences development and behavior in autism

by modulating mitochondrial physiology during prenatal development

^1*^Richard E Frye, MD PhD; ^2^Janet. Cakir, PhD; ^3,4^Shannon Rose, PhD; ^3,5^Leanna Delhey, MS; ^3,4^Sirish C Bennuri, MS; ^3,4^Marie Tippett, BA; ^3,4^Stepan Melnyk, MD, PhD; ^3,4^S. Jill James, PhD; ^6^Raymond F. Palmer, PhD; ^7^Christine Austin PhD; ^7^Paul Curtin PhD; ^7^Manish Arora, PhD,

^1^Barrow Neurological Institute at Phoenix Children's Hospital, Phoenix, AZ; ^2^North Carolina State University, Raleigh, NC;  ^3^Arkansas Children’s Research Institute, ^4^Department of Pediatrics and ^5^College of Public Health, University of Arkansas for Medical Sciences, and ^5^Arkansas Children’s Hospital, Little Rock AR; ^6^Department of Family and Community Medicine, University of Texas Health Science Center, San Antonio, TX; ^7^Department of Environmental Medicine and Public Health, Icahn School of Medicine at Mount Sinai, New York, NY;

Running Head: Air Pollution Disrupts Mitochondrial Metabolism in Autism

Supplementary Tables: 8; Supplementary Figures: 3; Words 1,080

Revision: 2 (Version 2.1); Date: Friday, July 24, 2020

This study has not been published previously.

|  |
| --- |
| Figure S1. Page 1 of the Developmental and Neurobehavioral Regression (DANR) Questionnaire. Additional pages are completed for additional regressions. |

| Table. S1 Participant Characteristics | |  |  |
| --- | --- | --- | --- |
| Variable | Typically Developing (n=  6) | ASD without  Regression (n=51) | ASD with  Regression (n=45) |
| Age, mean (SD), years months | 8 y 6 m (2 y 6 m) | 10y 9m (3y 10m) | 10y 11m (3y 3m) |
| Females, N (%) | 3 (50%) | 17 (33%) | 6 (13%) |
| ASD Diagnostic Documentation, N (%) |  |  |  |
| Autism Diagnostic Observation Schedule |  | 9 (18%) | 17 (38%) |
| Autism Diagnostic Interview-Revised |  | 21 (41%) | 17 (38%) |
| Gold Standard Diagnosis |  | 30 (59%) | 32 (71%) |
| Diagnosis by physician, psychologist, and speech therapist (Arkansas State Standard) |  | 12 (24%) | 14 (31%) |
| DSM diagnosis by physician with standardized, validated questionnaires & diagnosis confirmation by the Principal Investigator |  | 37 (73%) | 30 (67%) |
| Regression, N (%) |  |  | 45 (47%) |
| Single Regression |  |  | 23 (51%) |
| Age at 1^st^ Regression |  |  | 20m (8m) |
| Lost of Language Skills |  |  | 42 (93%) |
| Lost of Social Skills |  |  | 34 (76%) |
| Lost of Gross Motor Skills |  |  | 23 (51%) |
| Lost of Fine Motor Skills |  |  | 25 (56%) |
| Associated Fever |  |  | 19 (42%) |
| Associated Illness |  |  | 21 (47%) |
| Associated Seizure |  |  | 14 (31%) |
| Neurodevelopmental and Behavior Score  Mean (St Dev) |  |  |  |
| Vineland Adaptive Behavior Scale  (Scaled Score) |  |  |  |
| - Communication Subscale | 109 (10) | 70 (17) | 65 (20) |
| - Daily Living Skills Subscale | 101 (16) | 69 (18) | 65 (15) |
| - Social Subscale | 112 (15) | 67 (17) | 62 (13) |
| - Motor Subscale | 106 (9) | 80 (22) | 74 (12) |
| - Adaptive Behavioral Composite | 109 (13) | 67 (17) | 62 (14) |
| Social Responsiveness Scale (T-Score) |  |  |  |
| - Awareness | 48 (7) | 74 (13) | 77 (11) |
| - Cognitive | 46 (5) | 79 (13) | 79 (10) |
| - Communication | 46 (5) | 78 (13) | 83 (9) |
| - Motivation | 49 (8) | 70 (14) | 74 (13) |
| - Mannerisms | 45 (2) | 79 (17) | 81 (11) |
| - Total | 46 (3) | 81 (13) | 84 (9) |
| Aberrant Behavior Checklist (Raw Score) |  |  |  |
| - Irritability | 2 (3) | 15 (9) | 14 (8) |
| - Lethargy / Social Withdrawal | 2 (2) | 11 (8) | 12 (8) |
| - Stereotyped Movements | 0 (0) | 6 (5) | 6 (5) |
| - Hyperactivity | 4 (7) | 22 (11) | 22 (12) |
| - Inappropriate Speech | 0 (1) | 4 (3) | 4 (4) |
| - Total | 6 (10) | 54 (31) | 58 (17) |
| Comorbid Conditions (Parent Report), N (%) |  |  |  |
| Neurologic | 1 (17%) | 43 (84%) | 35 (78%) |
| Allergic | 2 (33%) | 29 (57%) | 31 (69%) |
| Psychiatric | 0 (0%) | 34 (67%) | 27 (60%) |
| Gastrointestinal | 2 (33%) | 37 (73%) | 38 (84%) |
| Immune | 2 (33%) | 38 (75%) | 42 (93%) |
| Growth | 1 (17%) | 20 (39%) | 17 (38%) |
| Endocrine | 0 (0%) | 5 (10%) | 5 (11%) |
| Cardiovascular | 0 (0%) | 3 (6%) | 7 (16%) |
| Comorbid Conditions (Medical Records), N (%) |  |  |  |
| Food Allergies/Intolerances | 1 (17%) | 33 (65%) | 35 (78%) |
| Epilepsy | 0 (0%) | 13 (25%) | 11 (24%) |
| Chronic Constipation | 0 (0%) | 19 (37%) | 17 (38%) |
| Fatigue/Exercise Intolerance | 0 (0%) | 14 (27%) | 8 (17%) |
| Recurrent Infections (AAAAI Criteria) | 0 (0%) | 10 (20%) | 8 (18%) |
| Gross Motor Delay | 0 (0%) | 28 (54%) | 31 (67%) |
| Hypogammaglobinemia | 0 (0%) | 6 (12%) | 6 (13%) |
| Failure to Thrive | 0 (0%) | 6 (12%) | 8 (17%) |
| Genetic Syndrome | 0 (0%) | 5 (10%) | 1 (2%) |
| Treatments, N (%) |  |  |  |
| Gastrointestinal Medications | 2 (33%) | 24 (47%) | 19 (42%) |
| Melatonin | 0 (0%) | 20 (39%) | 19 (42%) |
| Allergy/Asthma Medications | 0 (0%) | 12 (24%) | 18 (40%) |
| Mineral Supplements | 0 (0%) | 17 (33%) | 10 (22%) |
| Antiepileptic Medications | 0 (0%) | 7 (14%) | 15 (33%) |
| Antimicrobial Medications | 0 (0%) | 8 (16%) | 13 (29%) |
| Other Psychotropic Medications | 0 (0%) | 9 (18%) | 10 (22%) |
| Immunomodulatory Medications | 0 (0%) | 9 (18%) | 7 (16%) |
| Stimulant | 0 (0%) | 11 (22%) | 5 (11%) |
| Alpha-adrenergic agonists | 0 (0%) | 4 (8%) | 9 (20%) |
| Selective Serotonin Reuptake Inhibitors | 0 (0%) | 7 (14%) | 3 (7%) |
| Hormone Supplementation | 0 (0%) | 2 (4%) | 5 (11%) |
| Thyroid Supplementation | 0 (0%) | 3 (6%) | 3 (7%) |
| Beta Blocker | 0 (0%) | 3 (6%) | 1 (2%) |
| Dietary Formula | 0 (0%) | 1 (2%) | 0 (0%) |

**RESULTS**

3.1 Air Pollution Exposure, Mitochondrial Function and Redox Metabolism Between Groups

| Table S2. Basic Statistics [Mean (SD)] for Prenatal PM2.5 Exposure, Mitochondrial Parameters and Redox Metabolism by Group | | | | | | | | |
| --- | --- | --- | --- | --- | --- | --- | --- | --- |
|  | ^ave^PM_2.5_ | ^max^PM_2.5_ | ^ALR^ | ^PLR^ | ^MRC^ | ^RC^ | ^iGSH^ | ^IGSSG^ |
| Overall ASD (n=96) | 12.2  (2.4) | 31.0  (8.6) | 31.7 (15.7) | 11.1 (13.9) | 109.5 (94.0) | 76.2 (54.6) | 24.1 (3.8) | 0.6 (0.1) |
| Without Regression (n=51) | 12.4  (2.0) | 31.1  (7.0) | 31.1 (16.4) | 9.9 (15.8) | 109.4 (70.8) | 76.9 (56.7) | 24.1 (3.6) | 0.6 (0.1) |
| With Regression (n=45) | 12.1  (2.8) | 32.0  (10.0) | 32.6 (14.8) | 12.5 (11.1) | 109.5 (64.6) | 75.2 (65.5) | 24.0 (4.0) | 0.6 (0.1) |
| Overall TD (n=6) | 10.4  (1.5) | 24.5  (5.2) | 31.1 (12.0) | 11.8 (7.3) | 103.5 (47.1) | 69.9 (39.4) | 23.4 (3.0) | 0.6 (0.1) |
| TD Siblings (n=3) | 10.3  (2.0) | 23.9  (5.6) | 25.8 (26.4) | 9.6 (7.3) | 97.6 (32.7) | 68.1 (28.1) | 23.6 (2.7) | 0.7 (0.1) |
| TD Non-siblings (n=3) | 10.5  (1.1) | 25.1  (6.0) | 39.6 (40.7) | 15.4 (5.9) | 112.7 (64.5) | 72.9 (54.1) | 23.2 (4.0) | 0.6 (0.1) |

Adenosine triphosphate Linked Respiration (ALR), Proton Leak Respiration (PLR), Maximal Reserve Capacity (MRC) and Reserve Capacity (RC); intracellular reduced glutathione (GSH); intracellular oxidized glutathione (GSSG)

Individuals with ASD demonstrated higher ^max^PM_2.5_ and ^ave^PM_2.5_ values that TD individuals (Table 2) consistent with previous studies but this difference was only borderline significant [F(1,101)=3.32, p=0.07, *d’* = 0.36 and F(1,101) = 3.35, p=0.07. *d’* = 0.36] most likely due to the small TD sample size (See Table S1).

The average respiratory parameters for the ASD were not significantly different than the control group but TD siblings demonstrated a significantly lower Adenosine Triphosphate (ATP) Linked Respiration (ALR) [F(1,29)=13.64,p<0.001, *d’* = 1.34] and Proton Leak Respiration (PLR) [F(1,29)=5.24, p<0.05, *d’* = 0.84] as compared to non-sibling TD controls (See Table S2). The average intracellular reduced glutathione (iGSH) measures were not significantly different between the ASD and control groups.

Effect of Neurodevelopmental Regression

Similar ^max^PM_2.5_ and ^ave^PM_2.5_ was found between those with or without a history of NDR. Respiratory measures of ATP production were not different between those with or without a history of NDR but PLR was higher in those with a history of NDR as compared to those without a history of NDR [F(1,463)=3.82,p=0.05, *d’* = 0.18] (See Table S2). There is no significant difference in neurodevelopment or behavior between those with or without a history of NDR. There was no significant difference in iGSH or intracellular oxidized glutathione (iGSSG) between those with or without a history of NDR.

The Effect of Prenatal Air Pollution

*Mitochondrial Respiration:* The relationship between mitochondrial function and both ^max^PM_2.5_ and ^ave^PM_2.5_ depended on the history of NDR as demonstrated by statistically signficant interactions between Adenosine triphosphate Linked Respiration (ALR), Proton Leak Respiration (PLR), Maximal Reserve Capacity (MRC) and Reserve Capacity (RC) and NDR for both and ^max^PM_2.5_  [ALR: F(1,461)=23.80, p<0.0001; PLR F(1,460)=15.50, p<0.0001; Age F(1,460)= 8.31,p<0.01; MRC: F(1,461)=23.89, p<0.0001; RC: F(1,461)=21.66, p<0.0001] and  ^ave^PM_2.5_ [ALR: F(1,461)=23.44,p<0.0001; PLR F(1,460)=16.71, p<0.0001; Age F(1,460)= 8.36,p<0.01; MRC: F(1,461)=19.52,p<0.0001; RC: F(1,461)=14.90,p=0.0001].

Overall, for those with a history of NDR, higher prenatal ^max^PM_2.5_ [ALR: F(1,208)=19.76, p<0.0001; PLR: F(1,208)=39.50,p=0.001; MRC: F(1,208)=16.01, p<0.0001; RC: F(1,208)=13.83,p<0.001] and higher ^ave^PM_2.5_ [ALR: F(1,208)=20.98, p<0.0001; PLR: F(1,208)=21.42, p<0.0001; MRC: F(1,208)=20.82, p<0.0001; RC: F(1,207)=21.73, p<0.0001, Age F(1,207)=4.92,p<0.05] was related to higher mitochondrial respiration. While for those without a history of NDR, higher prenatal ^max^PM_2.5_ [ALR: F(1,252)=10.28, p=0.001, Age F(1,252)=4.15,p<0.05; PLR(1,252)=3.11, p=0.08, Age F(1,252)=9.93, p=0.001; MRC: F(1,252)=12.549, p<0.001, Age F(1,252)=5.04,p<0.05; RC: F(1,252)=12.02, p<0.001, Age F(1,252)=5.38, p<0.05] and higher ^ave^PM_2.5_ [ALR: F(1,252)=9.89, p=0.001, Age F(1,252)=4.68,p<0.05; PLR(1,252)=10.48, p=0.001, Age F(1,252)=13.02, p<0.001; MRC: F(1,252)=12.54, p<0.001, Age F(1,252)=5.04, p<0.05; RC: F(1,252)=5.36, p<0.05, Age F(1,252)=4.70,p<0.05] was related to lower mitochondrial respiration (See Figure 1).

*Behavior:* The relationship between prenatal ^max^PM_2.5_ and Adaptive Behavioral Composite of the Vineland Adaptive Behavior Scale (VABS) a measure of overall functional abilities, was influenced by a history of NDR [F(1,108)=5.29, p<0.05]. Higher prenatal ^max^PM_2.5_ exposure was related to a lower VABS score [F(1.57)=7.18, p<0.01] for those without a history of NDR but no significant relationship was found for those with a history of NDR. VABS was not statistically significantly related to prenatal ^ave^PM_2.5_.

The Social Responsiveness Scale (SRS), an index of ASD symptoms, was found to be related to prenatal ^ave^PM_2.5_ such that a higher prenatal ^ave^PM_2.5_ was associate with a lower SRS score [F(1,107)=7.75, p<0.01] when account for the influence of VABS score [F(1,107)=48.81, p<0.0001] regardless of the NDR history.

NDR influenced the relationship between Aberrant Behavior Checklist (ABC), measure of disruptive behavior and ^ave^PM_2.5_ [F(1,107)=4.57, p=0.01; VABS F(1,107) = 19.87, p <0.0001] such that higher ABC scores were associated with higher ^ave^PM_2.5_ for those without a history of NDR while lower ABC scores were associated with higher ^ave^PM_2.5_ for those with a history of NDR although neither of these relationships were statistically significant when examined separately.

The Relationship of Mitochondrial Respiration to Redox Metabolism.

Intracellular reduced glutathione (GSH) was significantly related to respiratory parameters associated with ATP production but not associated with PLR. Higher ALR [F(1,463)=5.95,p=0.01], MRC [F(1,463)=7.71,p<0.01] and RC [F(1,463)=7.39,p<0.01] was related to lower intracellular GSH. Lower intracellular oxidized glutathione (GSSG) was associated with higher MRC [F(1,463)=4.58,p<0.05] and RC [F(1,463)=5.25,p<0.05]. The relationship between GSSG and PLR was dependent on a history of NDR [F(1,460)=4.70, p<0.05]. This interaction was due to a positive relationship between GSSG and PLR for those without NDR which was borderline significant [F(1,252)=2.91, p<0.10] but no significant relationship between GSSG and PLR for those with a history of NDR (See Supplemental Results).

The Relationship of Mitochondrial Respiration to Behavior.

Development and behavior were found to be related to mitochondrial function, although the relationship was slightly different depending on whether there was a history of NDR. The VABS was positively related to all respiratory measures, although the relation was influenced by NDR for ALR [F(1,433)=4.00, p<0.05]. The interactions were due to the fact that those without a history of NDR demonstrated a stronger relationship between VABS scores and ALR [F(1,226)=21.59, p<0.0001] than those with a history of NDR [ALR: F(1,207)=8.33, p<0.01, Age F(1,207)=24.91,p<0.0001]. For the remainder of the respiratory parameters, higher respiratory rates were associated with higher VABS scores (better development) [PLR: F(1,434)=15.56, p<0.0001; MRC: F(1,434) =26.27,p<0.0001; RC: F(1,434)=25.34,p<0.0001] with VABS scores lower for older children [PLR F(1,434)=10.56, p<0.01; MRC F(1,434)=9.63,p<0.01; RC F(1,434)=9.03,p<0.01].

The SRS was found to be associated with all measures of mitochondrial function although the relationship for ALR and MRC was different depending on a history of NDR [F(1,425)=5.67, p<0.05; MRC F(1,425)=4.21,p<0.05]. For those without a history of NDR, SRS score was not related to ALR or MRC whereas for those with a history of NDR SRS score decreased (less ASD symptoms) as both ALR [F(1,203)=21.34, p<0.0001] and MRC [F(1,203)=26.43,p<0.0001] increased and as VABS score increased [ALR F(1,203)=58.79, p<0.0001; MRC F(1,203)=56.23,p <0.0001]. SRS scores decreased (less ASD symptoms) as PLR [F(1,427)=8.18,p<0.01] and RC [F(1,427)=13.39, p<0.001] increased and VABS score increased [PLR: F(1,427)=152.97, p<0.0001; RC: F(1,427)=141.53,p<0.0001].

A lower ABC score was associated with higher PLR [F(1,435)=9.36, p<0.01] when accounting for the influence of VABS [F(1,435)=73.21,p<0.0001].

The Relationship between Redox Metabolism and Behavior.

The lower SRS score was related to a lower GSH concentration [F(1,107)=4.03,p<0.05] and a higher VABS score [F(1,107)=45.29,p<0.0001]. No other relationships were noted between redox metabolism and behavior.

Understanding the complex relationships: Mediation analysis

Given that development (VABS) and ASD symptoms (SRS, ABC) were influenced by mitochondrial function and mitochondrial function was influenced by air pollution, it is possible that the effect of air pollution on development and behavior may be indirect through the mitochondria or may be both direct and indirect when controlling for the effect of the mitochondria on behavior and development. Thus, mediation analysis using proc CAUSALMED in SAS was used to investigate these influences.

| Supplementary Table S3. Mediation Analysis for the Vineland Adaptive Behavior Scale | | | | | | |
| --- | --- | --- | --- | --- | --- | --- |
| VABS | Total Effect | Controlled Direct Effect | Natural Direct Effect | Natural Indirect Effect | % Mediated | % Eliminated |
| **ATP** |  |  |  |  |  |  |
| Preave | -0.7708* | -0.9584** | -0.9682** | 0.1974* | -25.6% | -24.3% |
| Premax | -0.2780** | -0.3146*** | -0.3146*** | 0.0366 | -13.1% | -13.1% |
| **Proton** |  |  |  |  |  |  |
| Preave | -0.7200* | -0.8133** | -0.8206** | 0.1007 | -14.0% | -13.0% |
| Premax | -0.2881*** | -0.3497*** | -0.3504*** | 0.0623** | -21.6% | -21.4% |
| **MRC** |  |  |  |  |  |  |
| Preave | -0.7252* | -0.9128** | -0.9128** | 0.1916* | -26.4% | -25.9% |
| Premax | -0.2769** | -0.3037*** | -0.3037*** | 0.0268 | -9.7% | -9.7% |
| **RC** |  |  |  |  |  |  |
| Preave | -0.7079* | -0.8784** | -0.8784** | 0.1716* | -24.2% | -24.1% |
| Premax | -0.2583** | -0.2828** | -0.2824** | 0.0241 | -9.3% | -9.4% |
|  |  |  |  |  |  |  |
| All / Sig Ave (SD) – ave |  |  |  |  | -22.5% (7.2%) /  -24.5% (2.1%) |  |
| All / Sig Ave (SD) – max |  |  |  |  | -21.6% (--) /  13.4% (5.7%) |  |
| ATP Para  Ave (SD) - ave |  |  |  |  | -24.5% (2.1%) |  |
| ATP Para  Ave (SD) - max |  |  |  |  | -10.7% (2.1%) |  |

*P<=0.05; **P<=0.01; ***P<=0.001

| Supplementary Table S4. Mediation Analysis for the Social Responsiveness Scale | | | | | | |
| --- | --- | --- | --- | --- | --- | --- |
| SRS | Total Effect | Controlled Direct Effect | Natural Direct Effect | Natural Indirect Effect | % Mediated | % Eliminated |
| **ATP** |  |  |  |  |  |  |
| Preave | -0.9219*** | -0.8627*** | -0.8437*** | -0.0782* | 8.5% | 6.4% |
| Premax | -0.1479** | -0.1302** | -0.1288** | -0.0191* | 12.9% | 12.0% |
| **Proton** |  |  |  |  |  |  |
| Preave | -1.1209*** | -1.0795*** | -1.0746*** | -0.0462 | 4.1% | 3.7% |
| Premax | -0.1722*** | -0.1477** | -0.1472** | -0.0250* | 14.5% | 14.3% |
| **MRC** |  |  |  |  |  |  |
| Preave | -0.9383*** | -0.8475*** | -0.8314*** | -0.1069** | 11.4% | 9.7% |
| Premax | -0.1544** | -0.1331** | -0.1320** | -0.0224* | 14.5% | 13.8% |
| **RC** |  |  |  |  |  |  |
| Preave | -0.9408*** | -0.8539*** | -0.8397*** | -0.1011** | 10.7% | 9.2% |
| Premax | -0.1564** | -0.1358** | -0.1349** | -0.0215* | 13.8% | 13.1% |
|  |  |  |  |  |  |  |
| All / Sig Ave (SD) – ave |  |  |  |  | 8.7% (3.3%) / 10.2% (1.5%) |  |
| All / Sig Ave (SD) – max |  |  |  |  | 13.9% (0.8%) / 13.9% (0.8%) |  |
| ATP Para  Ave (SD) - ave |  |  |  |  | 10.2% (1.5%) |  |
| ATP Para  Ave (SD) - max |  |  |  |  | 13.7% (0.8%) |  |

*P<=0.05; **P<=0.01; ***P<=0.001

| Supplementary Table S5. Mediation Analysis for the Aberrant Behavior Checklist | | | | | | |
| --- | --- | --- | --- | --- | --- | --- |
| ABC | Total Effect | Controlled Direct Effect | Natural Direct Effect | Natural Indirect Effect | % Mediated | % Eliminated |
| **Proton** |  |  |  |  |  |  |
| Preave | -0.00944 | 0.1293 | 0.1293 | -0.1388 | 1469% | 1469% |
| Premax | 0.0533 | 0.1336 | 0.1336 | -0.0803 | -150% | -150% |

Understanding the complex relationships: Structural equation modeling (SEM) analysis

| **Supplementary Table S6.** Structural equation modeling (SEM) analysis for those with neurodevelopmental regression. Model structure is outlined in Figure 2A. Four models were fit using each measure of Adenosine Triphosphate (ATP) production: ATP-Linked Respiration (ALR), Maximal Respiratory Capacity (MRC) and Reserve Capacity (RC). For each measure of ATP production models were fit with two measures of Autism Spectrum Disorder (ASD) behavior as measured by the Social Responsiveness Scale (SRS) and Aberrant Behavior Checklist (ABC) and two measures of air pollution, average prenatal PM_2.5_ exposure (Ave) and maximum PM_2.5_ exposure (Max). Also included in the model is Proton Leak Respiration (PLR), reduced intracellular glutathione (GSH), oxidized intracellular glutathione (GSSG) and age at which regression occurred (Age_reg_). The left five columns represent the fit statistics which are bolded and italic if they exceed the optimal value [CFI: Comparative Fit Index (Optimal ≥ 0.90); GH: Gamma Hat (Optimal ≥ 0.90); RMSEA: Root Mean Square Error of Approximation (Optimal ≤ 0.08); SRMR: Standardized Root Mean Square Residual (Optimal ≤ 0.08); VMS: Number of variables misspecified per power analysis]. The remaining columns outline the coefficients (first line) of the effects of one variable (from) on a second variable (to) as well as the proportion of the total effect (second line). Significance levels for coefficients are as follow: *p<=0.05; **p<=0.01; ***p<=0.001; Lastly, the last two columns outline the covariance values of the model. | | | | | | | | | | | | | | | | | |
| --- | --- | --- | --- | --- | --- | --- | --- | --- | --- | --- | --- | --- | --- | --- | --- | --- | --- |
|  | Model Diagnostic Statistics | | | | | To | PLR | Mito | ASD Behavior | | Development (Dev) | | | | GSH | Covariance | |
|  | CFI | GH | RMSEA | SRMR | VMS | From | PM_2.5_ | PLR | Dev | PM_2.5_ | Mito | PM_2.5_ | Age_reg_ | GSH | GSSG | ATP-PLR | PM-GSH |
| ALR |  |  |  |  |  |  |  |  |  |  |  |  |  |  |  |  |  |
| SRS/Ave | ***0.97*** | ***0.98*** | ***0.06*** | ***0.07*** | ***0*** |  | 0.30^***^ | 0.76^***^ | -0.46^***^ | -0.33^***^ | 0.21^**^ | -0.20^**^ | 0.04^***^ | 0.16^*^ | 0.30^***^ | -0.182 | -0.104 |
|  |  |  |  |  |  |  |  |  | 58% | 42% | 34% | 33% | 7% | 26% |  |  |  |
| SRS/Max | ***0.92*** | ***0.95*** | 0.09 | ***0.08*** | ***0*** |  | 0.39^***^ | 0.76^***^ | -0.43^***^ | -0.29^***^ | 0.20^**^ | -0.15^*^ | 0.05^***^ | 0.16^*^ | 0.30^***^ | 0.024 | 0.075 |
|  |  |  |  |  |  |  |  |  | 60% | 40% | 36% | 27% | 9% | 29% |  |  |  |
| ABC/Ave | ***0.99*** | ***0.99*** | ***0.03*** | ***0.05*** | ***0*** |  | 0.31^***^ | 0.76^***^ | -0.40^***^ | -0.19^***^ | 0.21^**^ | -0.20^**^ | 0.04^***^ | 0.15^*^ | 0.28^***^ | -0.179 | -0.105 |
|  |  |  |  |  |  |  |  |  | 68% | 32% | 35% | 33% | 7% | 25% |  |  |  |
| ABC/Max | ***0.95*** | ***0.98*** | ***0.07*** | ***0.07*** | ***0*** |  | 0.39^***^ | 0.76^***^ | -0.38^***^ | -0.12^*^ | 0.20^**^ | -0.15^*^ | 0.05^***^ | 0.16^*^ | 0.28^***^ | -0.035 | 0.045 |
|  |  |  |  |  |  |  |  |  | 76% | 24% | 36% | 27% | 9% | 29% |  |  |  |
| MRC |  |  |  |  |  |  |  |  |  |  |  |  |  |  |  |  |  |
| SRS/Ave | ***0.94*** | ***0.98*** | ***0.07*** | ***0.07*** | ***0*** |  | 0.30^***^ | 0.64^***^ | -0.46^***^ | -0.33^***^ | 0.23^**^ | -0.20^**^ | 0.04^***^ | 0.15^*^ | 0.29^***^ | -0.281 | -0.104 |
|  |  |  |  |  |  |  |  |  | 58% | 42% | 37% | 32% | 6% | 24% |  |  |  |
| SRS/Max | 0.88 | ***0.96*** | 0.10 | 0.09 | ***0*** |  | 0.39^***^ | 0.64^***^ | -0.43^***^ | -0.29^***^ | 0.20^**^ | -0.15^*^ | 0.05^***^ | 0.16^*^ | 0.30^***^ | -0.023 | 0.075 |
|  |  |  |  |  |  |  |  |  | 60% | 40% | 36% | 27% | 9% | 29% |  |  |  |
| ABC/Ave | ***0.97*** | ***0.99*** | ***0.05*** | ***0.06*** | ***0*** |  | 0.31^***^ | 0.64^***^ | -0.40^***^ | -0.19^***^ | 0.23^***^ | -0.20^**^ | 0.04^***^ | 0.15^*^ | 0.28^***^ | -0.275 | -0.105 |
|  |  |  |  |  |  |  |  |  | 68% | 32% | 37% | 32% | 6% | 24% |  |  |  |
| ABC/Max | ***0.93*** | ***0.98*** | ***0.07*** | ***0.07*** | ***0*** |  | 0.39^***^ | 0.64^***^ | -0.38^***^ | -0.12^*^ | 0.21^**^ | -0.15^*^ | 0.05^***^ | 0.16^*^ | 0.28^***^ | -0.035 | 0.045 |
|  |  |  |  |  |  |  |  |  | 76% | 25% | 37% | 26% | 9% | 28% |  |  |  |
| RC |  |  |  |  |  |  |  |  |  |  |  |  |  |  |  |  |  |
| SRS/Ave | ***0.94*** | ***0.98*** | ***0.07*** | ***0.07*** | ***0*** |  | 0.30^***^ | 0.58^***^ | -0.46^***^ | -0.33^***^ | 0.23^*^*^ | -0.20^**^ | 0.04^***^ | 0.15^*^ | 0.29^***^ | -0.246 | -0.104 |
|  |  |  |  |  |  |  |  |  | 58% | 42% | 37% | 32% | 6% | 24% |  |  |  |
| SRS/Max | 0.88 | ***0.96*** | 0.10 | 0.09 | ***0*** |  | 0.39^***^ | 0.58^***^ | -0.43^***^ | -0.29^***^ | 0.21^**^ | -0.14^*^ | 0.05^***^ | 0.16^*^ | 0.29^***^ | -0.031 | 0.075 |
|  |  |  |  |  |  |  |  |  | 60% | 40% | 38% | 25% | 9% | 29% |  |  |  |
| ABC/Ave | ***0.97*** | ***0.99*** | ***0.04*** | ***0.06*** | ***0*** |  | 0.31^***^ | 0.58^***^ | -0.40^***^ | -0.19^***^ | 0.23^***^ | -0.20^**^ | 0.04^***^ | 0.15^*^ | 0.29^***^ | -0.241 | -0.105 |
|  |  |  |  |  |  |  |  |  | 68% | 32% | 37% | 32% | 6% | 24% |  |  |  |
| ABC/Max | ***0.92*** | ***0.98*** | ***0.07*** | ***0.07*** | ***0*** |  | 0.39^***^ | 0.58^***^ | -0.38^***^ | -0.12^*^ | 0.21^**^ | -0.14^*^ | 0.05^***^ | 0.15^*^ | 0.29^***^ | -0.041 | 0.045 |
|  |  |  |  |  |  |  |  |  | 76% | 24% | 38% | 25% | 9% | 27% |  |  |  |

| 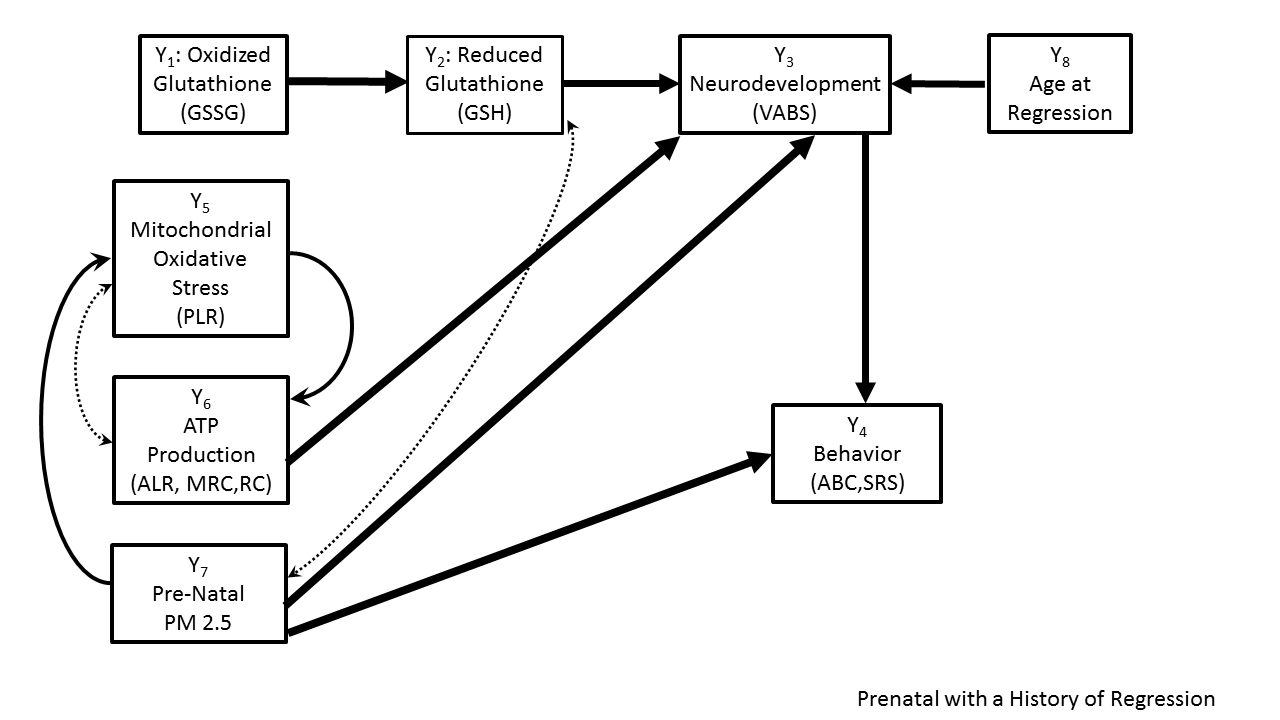 |
| --- |
| Supplementary Figure S2. Traditional structural equation model diagram. Solid lines depict direct influences while dotted lines depict modeled covariances. |

| **Supplementary Table S7.** Structural equation modeling (SEM) analysis for those without neurodevelopmental regression. Model structure is outlined in Figure 2B. Four models were fit using each measure of Adenosine Triphosphate (ATP) production: ATP-Linked Respiration (ALR), Maximal Respiratory Capacity (MRC) and Reserve Capacity (RC). For each measure of ATP production models were fit with two measures of Autism Spectrum Disorder (ASD) behavior as measured by the Social Responsiveness Scale (SRS) and Aberrant Behavior Checklist (ABC) and two measures of air pollution, average prenatal PM_2.5_ exposure (Ave) and maximum PM_2.5_ exposure (Max). Also included in the model is Proton Leak Respiration (PLR), reduced intracellular glutathione (GSH) and oxidized intracellular glutathione (GSSG). The left five columns represent the fit statistics which are bolded and italic if they exceed the optimal value [CFI: Comparative Fit Index (Optimal ≥ 0.90); GH: Gamma Hat (Optimal ≥ 0.90); RMSEA: Root Mean Square Error of Approximation (Optimal ≤ 0.08); SRMR: Standardized Root Mean Square Residual (Optimal ≤ 0.08); VMS: Number of variables misspecified per power analysis].The remaining columns outline the coefficients (first line) of the effects of one variable (from) on a second variable (to) as well as the proportion of the total effect (second line). Significance levels for coefficients are as follow: *p<=0.05; **p<=0.01; ***p<=0.001; Lastly, the last two columns outline the covariance values of the model. | | | | | | | | | | | | | | | | | | |
| --- | --- | --- | --- | --- | --- | --- | --- | --- | --- | --- | --- | --- | --- | --- | --- | --- | --- | --- |
|  |  |  |  |  |  | To | PLR | | Mito | | ASD Behavior | | Development (Dev) | | | GSH | Covariance | |
|  | CFI | GH | RMSEA | SRMR | VMS | From | GSH | GSSG | PLR | PM_2.5_ | Dev | PM_2.5_ | Mito | PM_2.5_ | GSH | GSSG | ATP-PLR | PM-GSH |
| ALR |  |  |  |  |  |  |  |  |  |  |  |  |  |  |  |  |  |  |
| SRS/Ave | ***0.96*** | ***0.98*** | ***0.08*** | ***0.06*** | ***0*** |  | -0.18^*^ | 0.20^**^ | 0.70^***^ | -0.06 | -0.52^***^ | -0.04 | 0.38^***^ | -0.31^**^ | 0.21^*^ | 0.34^***^ | -0.098 | 0.065 |
|  |  |  |  |  |  |  | 47% | 53% | 92% | 8% | 93% | 7% | 42% | 34% | 23% |  |  |  |
| SRS/Max | ***0.97*** | ***0.98*** | ***0.07*** | ***0.05*** | ***0*** |  | -0.18^*^ | 0.20^**^ | 0.69^***^ | -0.23^***^ | -0.48^***^ | -0.16 | 0.34^***^ | -0.40^***^ | 0.21^**^ | 0.34^***^ | -0.095 | 0.116 |
|  |  |  |  |  |  |  | 47% | 53% | 75% | 25% | 75% | 25% | 36% | 42% | 22% |  |  |  |
| ABC/Ave | ***0.99*** | ***0.99*** | ***0.01*** | ***0.05*** | ***0*** |  | -0.18^*^ | 0.20^**^ | 0.69^***^ | -0.06 | -0.22^***^ | 0.38^***^ | 0.38^***^ | -0.31^**^ | 0.21^*^ | 0.34^***^ | -0.098 | 0.065 |
|  |  |  |  |  |  |  | 47% | 53% | 92% | 8% | 37% | 63% | 42% | 34% | 23% |  |  |  |
| ABC/Max | ***0.99*** | ***0.99*** | ***0.02*** | ***0.04*** | ***0*** |  | -0.18^*^ | 0.20^**^ | 0.69^***^ | -0.23^***^ | -0.20^***^ | 0.36^***^ | 0.34^***^ | -0.40^***^ | 0.21^*^ | 0.34^***^ | -0.095 | 0.116 |
|  |  |  |  |  |  |  | 47% | 53% | 75% | 25% | 36% | 64% | 36% | 42% | 22% |  |  |  |
| MRC |  |  |  |  |  |  |  |  |  |  |  |  |  |  |  |  |  |  |
| SRS/Ave | ***0.95*** | ***0.97*** | ***0.07*** | ***0.06*** | ***0*** |  | -0.18^*^ | 0.20^**^ | 0.43^***^ | -0.06 | -0.52^***^ | -0.04 | 0.36^***^ | -0.32^***^ | 0.23^*^ | 0.33^***^ | -0.139 | 0.065 |
|  |  |  |  |  |  |  | 47% | 53% | 88% | 12% | 93% | 7% | 40% | 35% | 25% |  |  |  |
| SRS/Max | ***0.97*** | ***0.98*** | ***0.06*** | ***0.06*** | ***0*** |  | -0.18^*^ | 0.20^**^ | 0.42^***^ | -0.28^***^ | -0.48^***^ | -0.16 | 0.30^***^ | -0.40^***^ | 0.22^**^ | 0.33^***^ | -0.133 | 0.116 |
|  |  |  |  |  |  |  | 47% | 53% | 60% | 40% | 75% | 25% | 33% | 43% | 24% |  |  |  |
| ABC/Ave | ***0.99*** | ***0.99*** | ***0.02*** | ***0.05*** | ***0*** |  | -0.18^*^ | 0.20^**^ | 0.43^***^ | -0.06 | -0.22^***^ | 0.38^***^ | 0.38^***^ | -0.32^***^ | 0.23^**^ | 0.33^***^ | -0.139 | 0.065 |
|  |  |  |  |  |  |  | 47% | 53% | 88% | 12% | 37% | 63% | 41% | 34% | 25% |  |  |  |
| ABC/Max | ***1.00*** | ***0.99*** | ***0.00*** | ***0.05*** | ***0*** |  | -0.18^*^ | 0.20^**^ | 0.42^***^ | -0.29^***^ | -0.20^***^ | 0.36^***^ | 0.31^***^ | -0.40^***^ | 0.21^*^ | 0.33^***^ | -0.133 | 0.116 |
|  |  |  |  |  |  |  | 47% | 53% | 59% | 41% | 36% | 64% | 34% | 43% | 23% |  |  |  |
| RC |  |  |  |  |  |  |  |  |  |  |  |  |  |  |  |  |  |  |
| SRS/Ave | ***0.94*** | ***0.96*** | ***0.08*** | ***0.06*** | ***0*** |  | -0.18^*^ | 0.20^**^ | 0.34^***^ | -0.05 | -0.52^***^ | -0.04 | 0.36^***^ | -0.33^***^ | 0.22^*^ | 0.33^***^ | -0.135 | 0.065 |
|  |  |  |  |  |  |  | 47% | 53% | 87% | 13% | 93% | 7% | 40% | 36% | 24% |  |  |  |
| SRS/Max | ***0.97*** | ***0.98*** | ***0.06*** | ***0.06*** | ***0*** |  | -0.18^*^ | 0.20^**^ | 0.33^***^ | -0.28^***^ | -0.48^***^ | -0.16 | 0.30^***^ | -0.40^***^ | 0.21^**^ | 0.33^***^ | -0.128 | 0.116 |
|  |  |  |  |  |  |  | 47% | 53% | 54% | 46% | 75% | 25% | 33% | 44% | 23% |  |  |  |
| ABC/Ave | ***0.98*** | ***0.98*** | ***0.04*** | ***0.06*** | ***0*** |  | -0.18^*^ | 0.20^**^ | 0.34^***^ | -0.05 | -0.22^***^ | 0.38^***^ | 0.35^***^ | -0.33^***^ | 0.22^**^ | 0.33^***^ | -0.135 | 0.065 |
|  |  |  |  |  |  |  | 47% | 53% | 87% | 13% | 37% | 63% | 39% | 37% | 24% |  |  |  |
| ABC/Max | ***1.00*** | ***0.99*** | ***0.01*** | ***0.05*** | ***0*** |  | -0.18^*^ | 0.20^**^ | 0.33^***^ | -0.28^***^ | -0.20^***^ | 0.36^***^ | 0.30^***^ | -0.40^***^ | 0.21^*^ | 0.33^***^ | -0.128 | 0.116 |
|  |  |  |  |  |  |  | 47% | 53% | 54% | 46% | 36% | 64% | 33% | 44% | 23% |  |  |  |

| 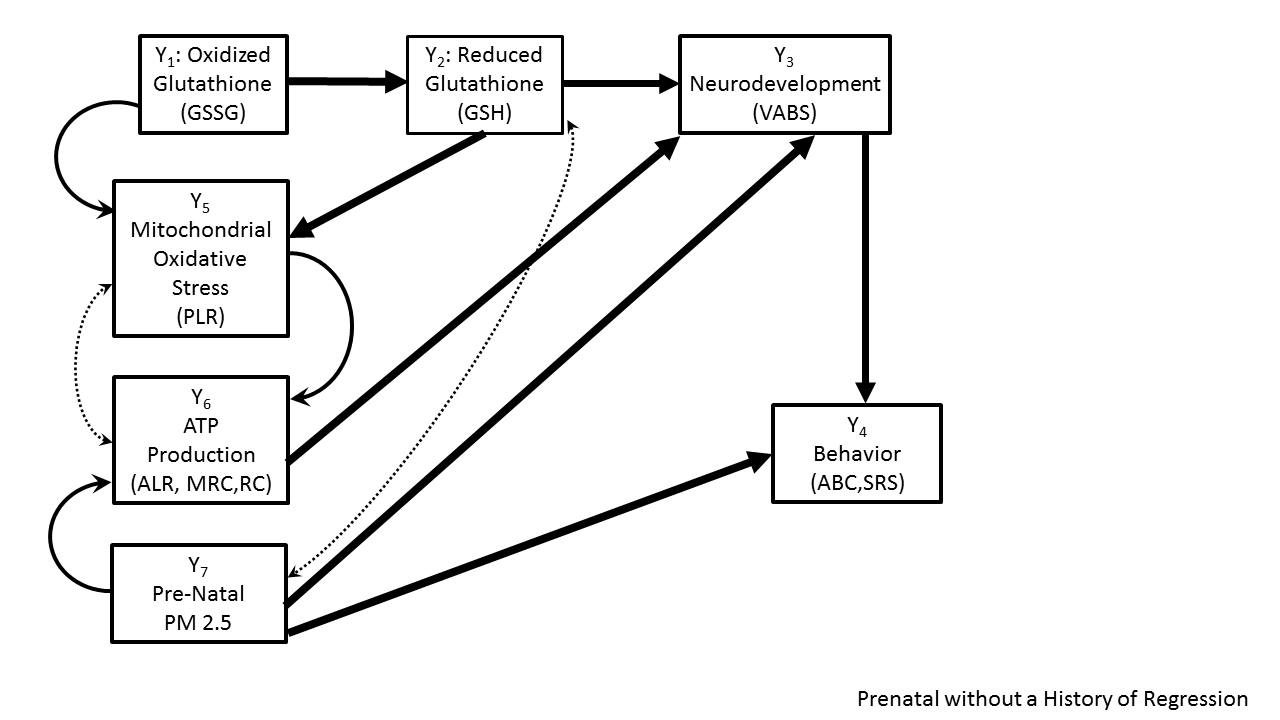 |
| --- |
| Supplementary Figure S3. Traditional structural equation model diagram. Solid lines depict direct influences while dotted lines depict modeled covariances. |

| Supplementary Table S8. Model Parameter Comparison. Comparison between model from children with autism spectrum disorder and neurodevelopmental regression as compared to those without neurodevelopmental regression. Z-scores from the parameters that were common across both models were subtracted and the two-tailed p-value based on the z distribution was calculated. | | | | | | | |
| --- | --- | --- | --- | --- | --- | --- | --- |
| To | Mito | Behavior | | Neurodevelopment (VABS) | | | GSH |
| From | Proton | Develop | PM2.5 | Mito | PM2.5 | GSH | GSSG |
| ATP |  |  |  |  |  |  |  |
| SRS/Ave | -1.37 | -1.20 | 4.19*** | 2.30 | -1.33 | 0.74 | 0.49 |
| SRS/Max | -1.53 | -0.96 | 6.33*** | 1.91 | -2.96* | 0.58 | 0.56 |
| ABC/Ave | -1.42 | 3.29** | 8.58*** | 2.31 | -1.34 | 0.76 | 0.85 |
| ABC/Max | -1.57 | 3.28** | 7.18*** | 1.91 | -2.97* | 0.61 | 0.92 |
| Max |  |  |  |  |  |  |  |
| SRS/Ave | -3.30** | -1.20 | 4.19*** | 0.79 | -1.46 | 0.98 | 0.49 |
| SRS/Max | -3.50** | -0.96 | 6.28*** | 1.36 | -2.98* | 0.73 | 0.57 |
| ABC/Ave | -3.32** | 3.26** | 8.57*** | 1.78 | -1.46 | 0.98 | 0.69 |
| ABC/Max | -3.52** | 3.25** | 7.13*** | 1.34 | -3.00* | 0.75 | 0.76 |
| Reserve |  |  |  |  |  |  |  |
| SRS/Ave | -8.22*** | -1.20 | 4.19*** | 1.72 | -1.62 | 0.94 | 0.51 |
| SRS/Max | -3.89*** | -0.96 | 6.28*** | 1.21 | -3.04* | 0.68 | 0.57 |
| ABC/Ave | -3.71** | 3.29** | 8.58*** | 1.73 | -1.64 | 0.94 | 0.66 |
| ABC/Max | -3.92*** | 3.28** | 7.13*** | 1.19 | -3.07* | 0.71 | 0.72 |

*p<=0.01; **p<=0.001; ***p<=0.0001
